# Changes in heme oxygenase level during development affect the adult life of *Drosophila melanogaster*

**DOI:** 10.3389/fncel.2023.1239101

**Published:** 2023-10-09

**Authors:** Bernadetta Bilska, Milena Damulewicz, Terence Al L. Abaquita, Elzbieta Pyza

**Affiliations:** Department of Cell Biology and Imaging, Institute of Zoology and Biomedical Research, Jagiellonian University, Cracow, Poland

**Keywords:** neurons, glia, cap’n’collar, survival, behavior, sleep, aging

## Abstract

Heme oxygenase (HO) has been shown to control various cellular processes in both mammals and *Drosophila melanogaster*. Here, we investigated how changes in HO levels in neurons and glial cells during development affect adult flies, by using the TARGET *Drosophila* system to manipulate the expression of the *ho* gene. The obtained data showed differences in adult survival, maximum lifespan, climbing, locomotor activity, and sleep, which depended on the level of HO (after *ho* up-regulation or downregulation), the timing of expression (chronic or at specific developmental stages), cell types (neurons or glia), sex (males or females), and age of flies. In addition to *ho,* the effects of changing the mRNA level of the *Drosophila* CNC factor gene (NRF2 homolog in mammals and master regulator of HO), were also examined to compare with those observed after changing *ho* expression. We showed that HO levels in neurons and glia must be maintained at an appropriate physiological level during development to ensure the well-being of adults. We also found that the downregulation of *ho* in either neurons or glia in the brain is compensated by *ho* expressed in the retina.

## 1. Introduction

Heme oxygenase (HO) maintains and modulates a broad spectrum of processes and among them, the most important ones are the degradation of intracellular heme and the subsequent generation of the products: carbon monoxide (CO), ferrous ion (Fe^2+^), and biliverdin (BV). Heme homeostasis is crucial for controlling cellular stress and processes modulated or dependent on it (Duvigneau et al., 2019).

In mammals, two active isoforms of HO have been identified: inducible HO-1 and constitutive HO-2, which differ in tissue distribution and function. The expression of HO-1 is detectable in most tissues, but it is exceptionally high in the spleen and liver. In contrast, HO-2 is abundant in the brain and testes and it is vital for their physiological functions (Ewing and Maines, 1997; Zakhary et al., 1997; Boehning and Snyder, 2003; Adachi et al., 2004; Alam et al., 2004; Mancuso, 2004; Andoh et al., 2006; Shibahara et al., 2007). The importance of HO-1 was demonstrated in the study using HO-1 knockout (HO-1-null) mice, which revealed its central role in development and iron homeostasis (Poss and Tonegawa, 1997a,b; Zhao et al., 2009; Kovtunovych et al., 2010). In humans, HO-1 deficiency results in early death, severe growth retardation, and multiple other disorders (Yachie et al., 1999; Kawashima et al., 2002; Radhakrishnan et al., 2011), confirming what has been found in animal studies.

In *Drosophila melanogaster*, the HO-1 homolog (dHO) is encoded only by a single gene (*ho*). HO in *Drosophila* has a unique structure and slower activity rate than mammalian and bacterial HOs (Zhang et al., 2004). It controls viability, development, iron accumulation, cell death (Cui et al., 2008), DNA damage signaling (Ida et al., 2013), protection against DNA damage caused by UV and white light (Damulewicz et al., 2017a,b), phototransduction, DNA repair, immune responses (Damulewicz et al., 2019), and circadian clock (Mandilaras and Missirlis, 2012; Klemz et al., 2017; Damulewicz et al., 2017a, 2019). The expression of *ho* is controlled by the circadian clock (Ceriani et al., 2002; Damulewicz et al., 2017a; Abaquita et al., 2021) and induced by many factors (Damulewicz et al., 2017b; Abaquita et al., 2021). Several processes, such as autophagy and apoptosis, have been reported to be associated and regulated by changes in the level of *ho* mRNA (Damulewicz et al., 2019; Abaquita et al., 2021, 2023). In all these studies *Drosophila* transgenic lines were used to induce higher or lower *ho* mRNA levels to identify HO biological functions. The genetic modifications employed in earlier studies were chronic, however, which means that changes in the *ho* expression were activated during embryonic development and maintained until adulthood. It has not been examined how modifications in *ho* expression at different developmental stages affect the life parameters of *Drosophila* adults.

Heme, the HO target for degradation, is a vital component for insect molting and metamorphosis. It is utilized as the prosthetic group of hemoproteins, including P450 enzymes involved in the synthesis of ecdysone and juvenile hormone (Feyereisen, 1999; Braz et al., 2001; Warren et al., 2002; Namiki et al., 2005; Rewitz et al., 2006; Chung et al., 2009; Iga and Kataoka, 2012; Sztal et al., 2012). Excessive heme degradation by sustained upregulation of HO in an anti-oxidative defense strategy may be detrimental to heme regulatory functions (Kimpara et al., 2000; Atamna et al., 2002; Zhu et al., 2002; Atamna, 2004; Sengupta et al., 2005; Smith et al., 2011). On the other hand, downregulation of *ho* can result in heme overload, which eventually promotes its abnormal binding and oxidative stress (Schmitt et al., 1993; Yoshida and Migita, 2000; Kikuchi et al., 2005; Kumar and Bandyopadhyay, 2005; Yang et al., 2008; Guenthner et al., 2009; Vallelian et al., 2015; Klemz et al., 2017; Chiabrando et al., 2018). Moreover, the dysfunction of heme metabolism may influence many molecular processes controlled by its activity-dependent end-products (Chiabrando et al., 2018; Duvigneau et al., 2019; Luu Hoang et al., 2021; Yang and Wang, 2022). Interestingly, the CNC factor, the *Drosophila* counterpart of the nuclear erythroid factor 2-related factor 2 (NRF2) that regulates HO-1, also plays an important role in the fly’s growth, development, and aging (Loboda et al., 2016).

In the nervous system, heme controls a variety of biological processes like energy production (Padmanaban et al., 1989; Sabová et al., 1993; Giraud et al., 1998; Mense and Zhang, 2006; Azuma et al., 2008; Kim et al., 2012), ion channels activity, gene expression, and miRNA processing (Smith et al., 2011). These processes are crucial for neuronal survival and differentiation (see Chiabrando et al., 2018). An imbalance in heme synthesis and degradation can mediate oxidative stress (Kagan et al., 2001; Klouche et al., 2004; Kumar and Bandyopadhyay, 2005; Schaer et al., 2013; Chiabrando et al., 2014; Wegiel et al., 2015), proteostasis failure (see Chiabrando et al., 2018), or mitochondrial decay and degeneration (Atamna, 2004). Increasing data show that both HO deficiency (see Mena et al., 2015) and overexpression (see Schipper et al., 1995, 2006; Song et al., 2007; Schipper et al., 2009; Ohnishi et al., 2010; Tronel et al., 2013; Barone et al., 2014; Chiang et al., 2018; Nitti et al., 2018; Waza et al., 2018; Schipper et al., 2019; Luu Hoang et al., 2021; Wu and Hsieh, 2022; Yang and Wang, 2022) are associated with neurodegenerative disorders. The brain is, therefore, at risk when HO is either at its high or low level. Moreover, there is no data about the effect of age-dependent (or stage-dependent) function of HO. In the present study we examined how manipulations of *ho* mRNA level at specific developmental stage affect adult flies. Knowing how HO is important for maintaining brain functions, we targeted separately neurons and glia, which play different functions in the brain and interact with each other.

We used the temporal and regional gene expression targeting (TARGET) system to manipulate gene expression spatially and temporally, and to do that a temperature-sensitive (ts) variant of the GAL80 protein was added to GAL4/UAS system. The GAL80ts molecule represses GAL4 at low temperatures, while at higher temperature GAL80ts is inactivated, which allows GAL4 to bind to UAS and to begin transcription of the target gene (McGuire et al., 2004). Here, the mRNA level of *ho* or *cnc* in the brain was modified chronically without GAL80ts or modified at specific developmental stages (either during larval, pupal, or adult life), using the tubulin promoter-GAL80ts transgene, and the longevity, fitness, and sleeping pattern of adult flies were examined.

The obtained results showed that changes in *ho* or *cnc* expression in the brain at different developmental stages adversely affect adult life compared to chronic expression. The influence of *ho* or *cnc* on the life expectancy, climbing performance, activity, and sleep pattern of *Drosophila* adults vary according to their expression level (upregulation or downregulation), cell types (neurons or glia), sex (males or females), and age of adult flies.

## 2. Materials and methods

### 2.1. Animals

The following *Drosophila* strains were used in the study: *repo*-Gal4 (pan-glial cell marker, BDRC no. 7415), *elav*-Gal4 (pan-neuronal cell marker), *tub*Gal80ts; *repo*-Gal4, *tub*Gal80ts; *elav*-Gal4, UAS-*ho* (Cui et al., 2008), UAS-*hoRNAi* (Cui et al., 2008), UAS-*cnc*, UAS-*cncRNAi*, UAS-*GFP.Valium10* (BDRC no. 35786), and wild type (CS). The strains UAS-*ho* and UAS-*hoRNAi* were kindly provided by Dr. Taketani (Kyoto Institute of Technology, Japan). Constructs are inserted on the 3rd chromosome in *w1118* background.

To study the effect of overexpression/silencing of *ho* or *cnc* at different developmental stages, flies crossed with strains containing *tub*Gal80ts were kept at different temperature conditions: (i) larva-specific, at 29°C from embryo to pupariation and then at 18°C; (ii) pupa-specific, at 18°C from embryo to pupariation followed by 29°C during the pupal stage and at 18°C after eclosion; (iii) adult-specific, at 18°C from embryo until eclosion and then at 29°C. For chronic overexpression/silencing, flies without *tub*Gal80ts were kept constantly at 25°C. Control flies, both parental strains backcrossed to wild-type flies, were kept in the same temperature conditions as the experimental flies.

### 2.2. Survival assay

After eclosion, flies were separated according to sex and kept in groups of 30 individuals in specific temperature conditions. Every day the number of dead flies was counted, and every 3 days, flies were transferred to the new vials with fresh food. The experiment continued until the death of the last fly in the vial.

### 2.3. Climbing assay (negative geotaxis test)

Males aged 7, 14, and 30 days-old were transferred into an empty vial without using CO_2_ to avoid effects of this type of anaesthesia on locomotion and climbing ability. After a short recovery, flies were gently tapped to the bottom of a vial, and those that climbed vertically beyond the 5 cm marked line in 15 s were counted. The experiment was carried out in a dark room with red light only, which is invisible to flies. Every trial with 30 flies was repeated three times. Every experimental group had three repetitions.

### 2.4. Locomotor activity and sleep

The locomotor activity was recorded using a *Drosophila* Activity Monitoring System (DAMS, Trikinetics). Males, 2 days-old, were anesthetized with CO_2_, placed on ice and then transferred into glass tubes (one fly per tube) sealed at both ends by food (agar, sugar, yeasts) and by a foam stopper. These tubes were placed in monitors (maximum 32 tubes per monitor) equipped with infrared light-emitting diodes and detectors that were connected to a computer. Whenever the fly passed the emitter/detector, the infrared beam was interrupted, transmitting a signal to the computer. To analyze the circadian period of locomotor activity, flies were maintained for 7 days under LD12:12 and next under constant darkness (DD) for 7 days. The locomotor activity was scored every 1 min. To study sleep, the activity of flies was analyzed on the second day of LD12:12 conditions. Sleep was measured as intervals of at least 5 min of inactivity.

Data were analyzed in Excel by using “Befly!” software (Department of Genetics, Leicester University). Lomb–Scargle normalized periodogram was used to determine rhythmic flies; flies with a period value lower than 10 (confidence level 0.05) were regarded as arrhythmic. Flies that did not survive until the end of each experiment were removed from analyses. Every experiment was repeated three times with at least 60 flies per group.

### 2.5. RNA extraction, cDNA synthesis, and qPCR

To compare *ho* or *cnc* expression between experimental groups, samples were collected at different developmental stages: the whole body of the 3rd instar larvae or pupae (5 individuals per sample, 3 repetitions), and heads of 5 days-old males and females (20 individuals per sample, 3 repetitions), separately. All samples were collected 1 h after the lights-on. We decided to examine gene expression in the whole body of larvae and pupae, and in heads of adults. In case of larvae we did not want to exclude motoneurons and glia located in the peripheral nervous system. There are about 700 neurons and 77 glial cells in each segment of the larval body (Bittern et al., 2021). Since we were not sure which cells in the central or peripheral nervous system were more affected in the experiments we decided to use whole body samples, although other tissues may have higher levels of both gene mRNA than neurons and glial cells. In case of pupae the nervous system is changed from larval to adult one and it is very difficult to dissect brains in early pupae. The experiments were carried out on wandering L3 larvae and in case of pupae in the pupal early stage when pupae changed color from white to brown (prepupae or P2 according to Bainbridge and Bownes, 1981). Total RNA was isolated using the Trizol method, purity was checked using NanoDrop and confirmed as acceptable with 260/280 and 260/230 ratios higher than 1.8. Total RNA (500 ng) was used for reverse transcription. Gene expression was measured using Kapa Sybr Green (KAPA Biosystems, Cape Town, South Africa) using specific primer sequences (*ho*: For 5′-ACCATTTGCCCGCCGGGATG-3′, Rev 5′-AGTGCGACGGCCAGCTTCCT-3′, gene accession no. CG14716; and *cnc*: For 5′-GAGGTGGAAATCGGAGATGA-3′, Rev 5′-CTGCTTGTAGAGCACCTCAGC-3′, gene accession no. CG43286) on StepOnePlus Real-Time PCR System. *Rpl32* was used as a reference gene (For 5′-TATGCTAAGCTGTCGCACAAATG-3′, Rev 5′-AGCACGTGTATAAAAAGTGCCA-3′, gene accession no. CG7939). A standard curve was used to calculate gene expression levels. The number of target gene copies was normalized to the reference gene.

### 2.6. Statistical analysis

All data were examined for distribution normality, and statistical tests were chosen accordingly. The Kaplan–Meier curve was used to present data on the survival percentage of flies plotted against their age (in days). The survival (in %) in every phase of the aging process and the maximum lifespan between genotypes (i.e., experimental group versus control 1 and experimental group versus control 2) were analyzed using the log-rank test performed with the R/R Studio free statistical version 4.2.0.^1^ The non-parametric analysis of variance (ANOVA) was used to analyse climbing performance and qPCR results (Dunn’s test). For locomotor activity and sleep analysis, the parametric ANOVA was used (Dunnett’s test). Statistical analyses were performed with the GraphPad Prism 7.05 software. Detailed statistics is provided in Supplementary Tables S1, S5, S7–S9.

## 3. Results

### 3.1. Effects of changing *ho* or *cnc* mRNA level in the brain at different developmental stages on the longevity of adult flies

**Figure 1:**
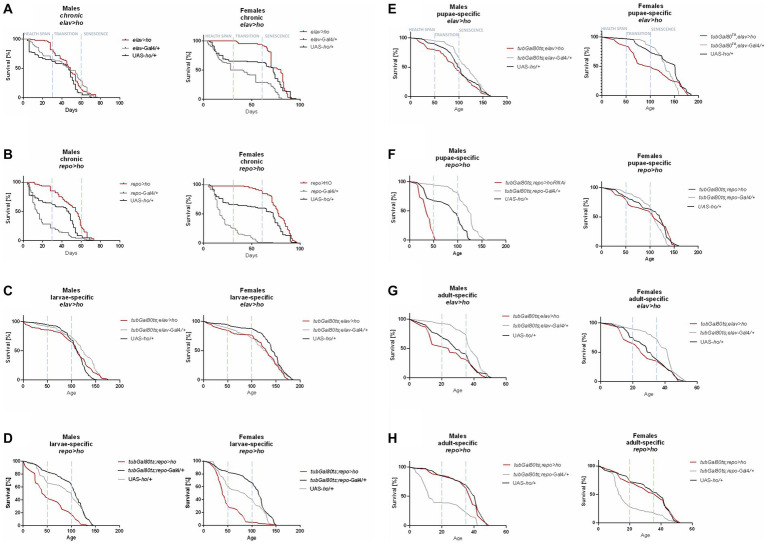
The survival curve of *Drosophila* adults is altered by *ho* overexpression in the brain (specifically in neurons or glia) at different developmental stages: chronic **(A,B)**, larva-specific **(C,D)**, pupa-specific **(E,F)**, and adult-specific **(G,H)**.

**Figure 2:**
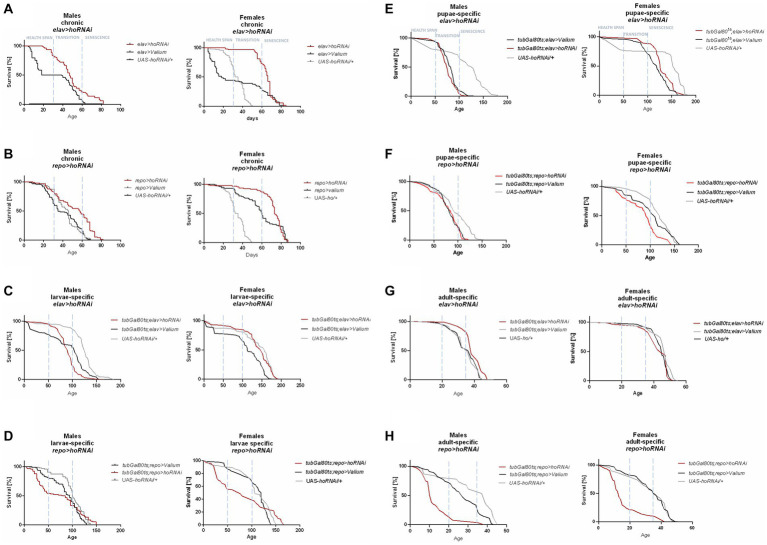
The survival curve of *Drosophila* adults is altered by *ho* silencing in the brain (specifically in neurons or glia) at different developmental stages: chronic **(A,B)**, larva-specific **(C,D)**, pupa-specific **(E,F)**, and adult-specific **(G,H)**.

The role of HO or its upstream regulator CNC in the brain during development was first examined on the survival pattern and maximum lifespan of adult flies. For developmental studies, the TARGET system is commonly used, however, flies are cultured at high or low temperatures which may affect their longevity. Universally, adult flies go through three phases of aging under an optimal temperature condition: health span (4–30 days after eclosion or DAE), transition phase (31–60 DAE), and senescence phase (61–120 DAE) (Phom et al., 2014). Here, we used 25°C as the optimal temperature for culturing of flies with chronic GAL4 activation, 18°C to inhibit Gal4 system, and 29°C to inactivate Gal80ts and activate cell-specific gene expression. For transgenic groups maintained at 18°C or 29°C, we used the following descriptions to identify the DAE range for each phase: (i) health span for those DAE with no natural death, (ii) transition phase for those DAE with a slight decline in the mortality curve showing 10% death, and (iii) senescence phase for those DAE with a steady decline in mortality curve represented by the window between the end of the transition phase till maximum lifespan of the fly (Ayajuddin et al., 2022). The DAE range for each phase could change after exposure to lower or higher temperature conditions than the optimal one (Mołoń et al., 2020). Effects of disrupting the expression of *ho* or *cnc* in the brain during development on the life expectancy of adults were analysed in each phase. In general, the adult longevity of *Drosophila* changed when *ho* or *cnc* mRNA level was changed during development. Interestingly, we observed opposite effects between chronic modification and those targeted at specific developmental stages (Table 1; Figures 1, 2; Supplementary Table S1). Survival was mostly better in adults when *ho* was either overexpressed or silenced chronically in the brain. Whereas low or unaffected survival was mainly detected when *ho* overexpression or silencing was explicitly done in the larval, pupal, or adult stage. We also observed differences in the effects of changing *ho* expression level in neurons or glia during development on adult longevity.

**Table 1:** Summary of the effects of *ho* mRNA level changes in neurons or glia at specific developmental stages on adult longevity, fitness and sleep.

Timing	HO level	Cell type	Life expectancy	Fitness	Sleep
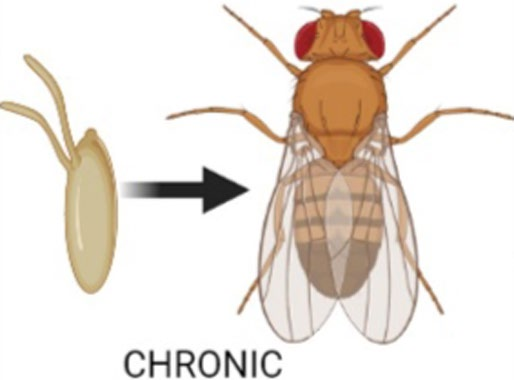	HO↑	Neurons	↑ survival in **young** ♂♀	No effect	↑ sleep in day
		Glia	↑ survival in **young** ♂♀ ↑ max lifespan in ♂♀	↓ climbing in **old** ♀ ↓ activity in **young** ♂	↑ sleep in day & night
	HO↓	Neurons	↑ survival in **very old** ♂ & **young** ♀ ↑ max lifespan in ♂♀	↓ climbing in **young** ♂♀ & **old** ♀ ↑ activity in **young** ♂	↓ sleep in day
		Glia	↑ survival in **old** ♂♀ ↑ max lifespan in ♂	↓ climbing in **old** ♂	No effect
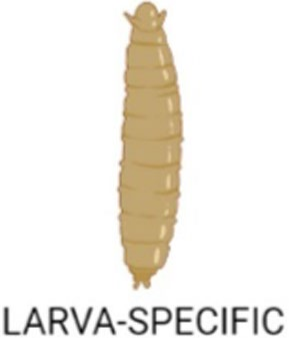	HO↑	Neurons	No effect	No effect	No effect
		Glia	↓ survival in **young to old** ♂♀ ↓ max lifespan in ♂♀	No effect	↓ sleep in day
	HO↓	Neurons	↓ survival in **old** ♂ ↓ max lifespan in ♂	No effect	No effect
		Glia	↓ survival in **young and very old** ♂♀	No effect	No effect
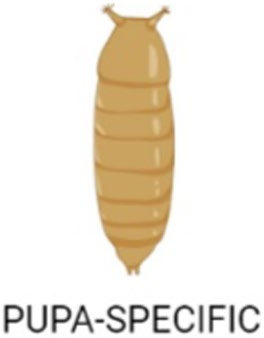	HO↑	Neurons	↓ survival in **young to old** ♀	No effect	No effect
		Glia	↓ survival in **young to old** ♂ ↓ max lifespan in ♂	No effect	No effect
	HO↓	Neurons	No effect	No effect	No effect
		Glia	↓ survival in **old** ♀ ↓ max lifespan in ♀	No effect	No effect
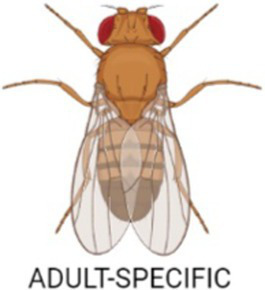	HO↑	Neurons	↓ survival in **old** ♂♀	No effect	No effect
		Glia	↓ survival in **very old** ♀	No effect	↑ sleep in day
	HO↓	Neurons	↑ survival in **old** ♂	No effect	No effect
		Glia	↓ survival in **young and very old** ♂♀ ↓ max lifespan in ♂♀	No effect	↑ sleep in day

Detailed statistics is described in Supplementary material.

Flies with chronic *ho* overexpression in neurons (*elav* > *ho*) had higher survival during the health span in both sexes, but over time, changes did not vary with the parental controls, except in females during senescence phase (Figure 1A). Similarly, chronic *ho* overexpression in glia (*repo* > *ho*) resulted in a higher percentage of survival during the health span stage for both male and female adults (Figure 1B). Upregulation of *ho* in neurons during the larval stage had the same effect (Figure 1C), while in glia, it resulted in a significantly lower chance of survival in the health span to the transition phase for both sexes which extended in males until senescence phase (Figure 1D). Pupa-specific *ho* overexpression in neurons resulted in a lower percentage of survival in the health span and the transition phase but only for females (Figure 1E), while in glia, a lower percentage of survival was observed in similar pattern but with male adults (Figure 1F). Activating *ho* overexpression in neurons during the adult stage resulted in lower survival during the health span in males only (Figure 1G), while in glia, adults did not have any changes in their survival pattern except for males in senescence phase (Figure 1H).

Chronic *ho* silencing either in neurons (*elav* > *hoRNAi*) or glia (*repo* > *hoRNAi*) produced similar survival patterns. Female adults survived better before senescence phase, while male adults had a higher percentage of survival during the senescence phase (Figures 2A,B). Larva-specific *ho* silencing in neurons did not give any significant differences in survival patterns, except in males with a lower percentage of survival during the transition phase (Figure 2C). On the contrary, larva-specific *ho* silencing in glia had lower survival during the health span and the senescence phase for both males and females (Figure 2D). The survival pattern of adults was not changed after downregulating *ho* expression in neurons during the pupal stage (Figure 2E), while in glia, only female adults showed a significantly lower chance of survival during the transition phase (Figure 2F). Silencing *ho* in neurons during the adult stage resulted in higher survival in the transition phase (only males) (Figure 2G). On the other hand, pan-glial *ho* silencing during the adult stage exhibited lower chances of survival during the health span and the senescence phase for both male and female adults (Figure 2H).

**Figure 3:**
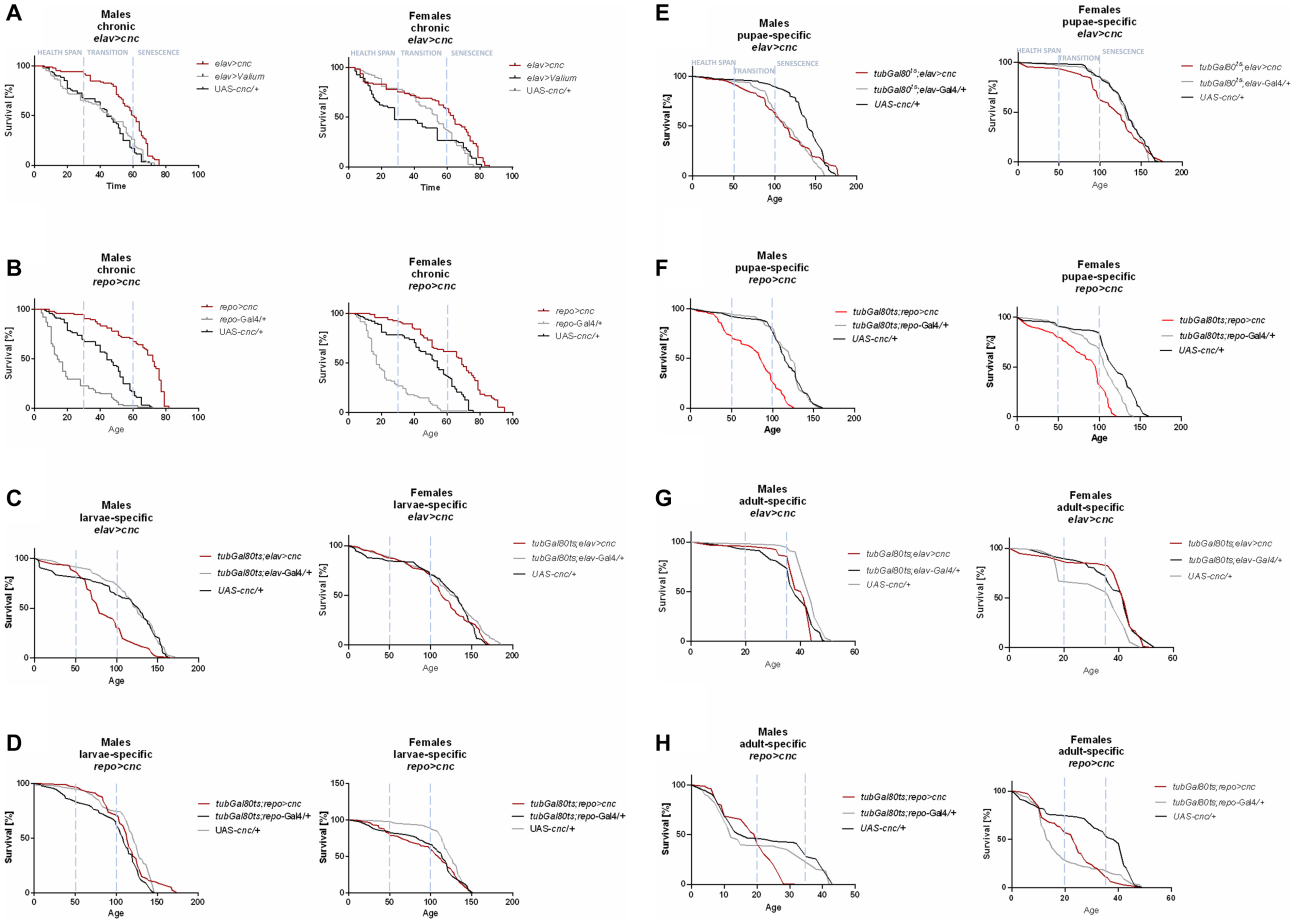
The survival curve of *Drosophila* adults is altered by *cnc* overexpression in the brain (specifically in neurons or glia) at different developmental stages: chronic **(A,B)**, larva-specific **(C,D)**, pupa-specific **(E,F)**, and adult-specific **(G,H)**.

Similarly, flies with modified *cnc* expression levels in the brain were observed to have opposite effects between chronic expression and those at specific developmental stages (Table 2). The survival of adults was better when *cnc* was either overexpressed or silenced continuously in comparison to those modifications activated specifically in the larval, pupal, or adult stage. The survival of chronic *elav* > *cnc* flies was significantly higher in male adults during health span while female adults during the senescence phase (Figure 3A). In chronic *repo* > *cnc* flies, the higher percentage of survival was almost detectable in all phases except for females in senescence stage (Figure 3B). Upregulation of *cnc* in glia during the larval stage did not produce any significant differences in survival patterns (Figure 3D), while in neurons, only males had lower percentages of survival during the transition to the senescence phase (Figure 3C). Pupa-specific *cnc* overexpression in neurons did not produce any significant differences, except in females with a lower percentage of survival during the transition phase (Figure 3E). On the contrary, female adults with pupa-specific *cnc* overexpression in glia had a lower percentage of survival in all phases (Figure 3F). Activating *cnc* overexpression in neurons during the adult stage did not produce any significant differences in survival patterns, except males in the senescence phase (Figure 3G), while in glia, a significantly lower survival was observed only in males during the transition phase (Figure 3H).

**Table 2:** Summary of the effects of *cnc* mRNA level changes in neurons or glia at specific developmental stages on adult longevity, fitness and sleep.

Timing	CNC level	Cell type	Life expectancy	Fitness	Sleep
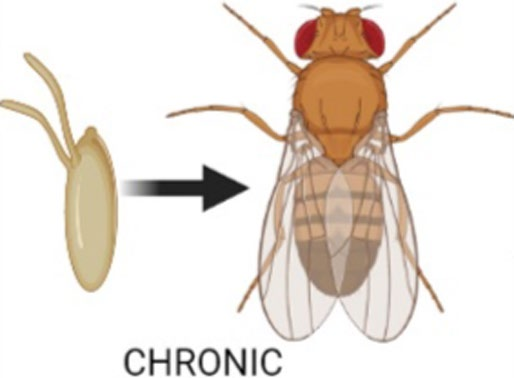	CNC↑	Neurons	↑ survival in **young** ♂ and **old** ♀ ↑ max lifespan in ♂♀	↓ climbing in **old** ♂	↑ sleep in day & night
		Glia	↑ survival in **young to old** ♂♀ ↑ max lifespan in ♂♀	↓ climbing in **old** ♀ ↓ activity in **young** ♂	↑ sleep in day & night
	CNC↓	Neurons	↑ survival in **young to very old** ♀ and **young** ♂ ↑ max lifespan in ♀	↓ activity in **young** ♂	↑ sleep in day & night
		Glia	↑ survival in **old** ♂♀	↓ activity in ♂	↑ sleep in day & night
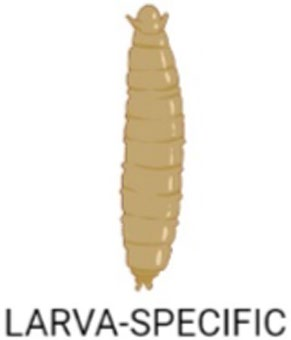	CNC↑	Neurons	↓ survival in **old to very old** ♂	No effect	No effect
		Glia	No effect	No effect	↓ sleep in night
	CNC↓	Neurons	↓ survival in **very old** ♀ ↓ max lifespan in ♂♀	No effect	↓ sleep in day & night
		Glia	↓ survival in **young** ♂ and **very old** ♀ ↓ max lifespan in ♂♀	No effect	↓ sleep in day & night
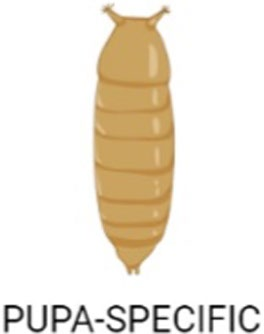	CNC↑	Neurons	↓ survival in **old** ♀	No effect	↑ sleep in day
		Glia	↓ survival in **young to very old** ♂♀ ↓ max lifespan in ♂♀	No effect	No effect
	CNC↓	Neurons	No effect	No effect	↓ sleep in night
		Glia	↓ survival in **young to very old** ♂ ↓ max lifespan in ♂	No effect	
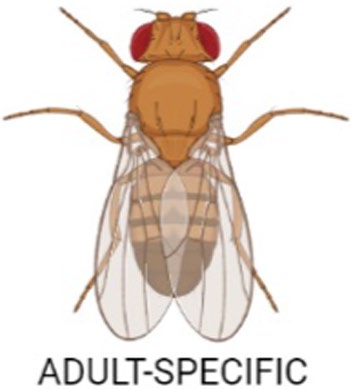	CNC↑	Neurons	↓ survival in **very old** ♂	No effect	No effect
		Glia	↓ survival in **old** ♂ ↓ max lifespan in ♂	No effect	↑ sleep in day
	CNC↓	Neurons	↓ survival in **old** ♂ and **very old** ♀	No effect	
		Glia	↓ survival in **very old** ♂♀ ↓ max lifespan in **old** ♂♀	No effect	

Detailed statistics is described in Supplementary material.

**Figure 4:**
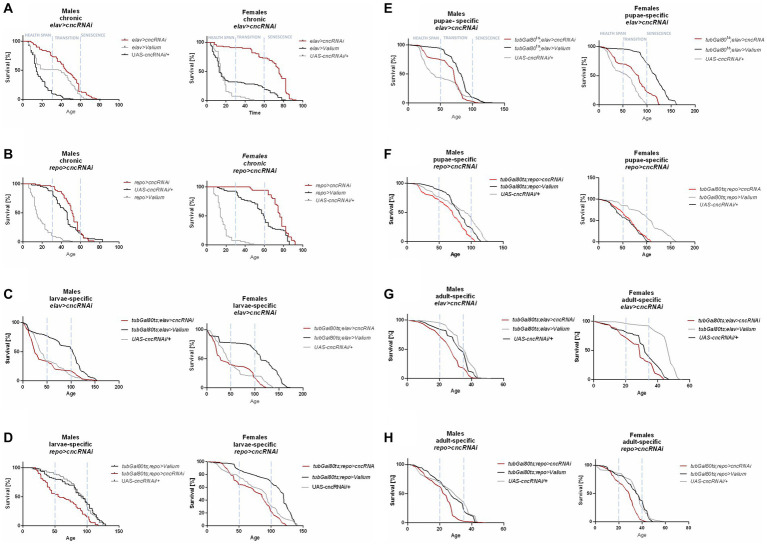
The survival curve of *Drosophila* adults is altered by *cnc* silencing in the brain (specifically in neurons or glia) at different developmental stages: chronic **(A,B)**, larva-specific **(C,D)**, pupa-specific **(E,F)**, and adult-specific **(G,H)**.

For chronic *elav* > *cncRNAi* flies, a higher percentage of survival was observed in all phases for female adults and only in the health span for males (Figure 4A). In chronic *repo* > *cncRNAi* flies, a better percentage of survival was detectable in the transition to the senescence phase for females only (Figure 4B). Larva-specific *cnc* silencing in neurons obtained a lower percentage of survival in the senescence phase (only females) (Figure 4C), whereas in glia, male adults had a lower percentage of survival in the health span stage and females in the senescence phase as well (Figure 4D). After downregulating *cnc* expression in the glia during the pupal stage, only males showed significantly lower survival in the transition to the senescence phase (Figure 4F). In contrast, *cnc* silencing in neurons at the pupal stage did not affect survival at all (Figure 4E). A significantly lower likelihood of survival was found during the transition phase for males and the senescence phase for females after silencing *cnc* in neurons during the adult stage (Figure 4G). In comparison, adult-specific pan-glial *cnc* silencing generated a lower percentage of survival only during the senescence phase for both sexes (Figure 4H).

The maximum lifespan of adults in each experimental condition reflected differences when the expression level of *ho* or *cnc* was targeted chronically or at a specific developmental stage (Supplementary Table S1). Under 25°C, the maximum lifespan in chronic experiments was 73–97 DAE. For adult-specific experiments, when flies were kept at 29°C during adult life, aging was faster (Mołoń et al., 2020). It may explain why adults survived only 32–52 DAE. For larva- and pupa-specific experiments that were kept at 18°C during their adult life, longevity generally increased (Mołoń et al., 2020). In this case, adults survived maximum 194 DAE. The effects of modifying *ho* expression level were not adequately strong to cause changes in the maximum lifespan of adult flies, regardless of the timing (chronically or at specific developmental stages) when overexpression/silencing was done. Most changes in the maximum lifespan were detected only in flies with *ho* silencing in the glia. Silencing of *ho* in the glia chronically, in pupae or adults did not increase survival compared with parental controls, while larva-specific *ho* silencing in the glia extended lifespan of adult flies. In the case of the chronic modifications in *cnc* expression, we found that flies with *cnc* overexpression in either neurons or glia survived significantly longer than controls. For flies with pan-neuronal overexpression at specific developmental stages, the flies treated during the adult stage showed a significantly shorter maximum lifespan compared to the control groups. It had a similar pattern with adult-specific *repo* > *cnc*. Also, both larva- and pupa-specific overexpression of *cnc* in glia did not survive longer than their parental controls. The downregulation of *cnc* in neurons or glia mostly led to a shorter maximum lifespan, regardless of which developmental stage was treated, except for chronic *elav* > *cncRNAi* which had an extended maximum lifespan. Differences in maximum lifespan between male and female adults were also observed. In most cases, females had longer maximum lifespans compared to males.

### 3.2. Effects of changing *ho* or *cnc* expression in the brain at different developmental stages on the fitness of adult flies

**Figure 5:**
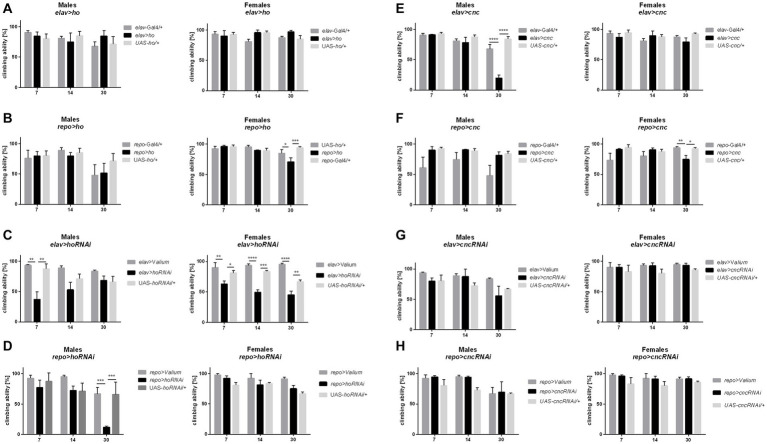
The climbing performance of *Drosophila* adults 7, 14, and 30 days after eclosion (DAE) was affected by chronic changes in *ho* **(A–D)** or *cnc* **(E–H)** expression in the brain, specifically in neurons or glia. Data are shown as means ± SD. Statistically significant differences between genotypes are indicated with *asterisks* (^*^p < 0.05, ^**^p < 0.01, ^***^p < 0.001, ^****^p < 0.0001).

Changing the *ho* or *cnc* mRNA level, specifically during the larval, pupal, or adult stage, did not cause any significant differences in the climbing performance of adult flies (Supplementary Figures S2–S4 and Supplementary Table S5). The effects of *ho* or *cnc* expression modifications on the climbing behavior of flies were visible only after chronic overexpression or silencing of either *ho* or *cnc* in the brain. Flies with sustained upregulation of *ho* expression, *elav* > *ho,* did not show any changes in their climbing ability (Figure 5A and Supplementary Table S4), while *repo* > *ho* had a significantly lower percentage of climbing at 30 DAE, which were observed only in female adults (Figure 5B). For *elav* > *hoRNAi* flies, climbing defects were observed in males at 7 DAE, whereas in females, the effect was stronger as their climbing performance at 7, 14, and 30 DAE was decreased (Figure 5C). In *repo* > *hoRNAi* flies, only males had a lower percentage of climbing at 30 DAE (Figure 5D). In flies with chronic *cnc* overexpression, sex-dependent effects were observed at 30 DAE. Only a few males of *elav* > *cnc* were able to climb up the 5 cm demarcation line (Figure 5E), while the same effect was observed in *repo* > *cnc* females (Figure 5F). Continuous *cnc* silencing in both neurons and glia did not bring any significant results regarding climbing performance (Figures 5G,H).

The locomotor activity of flies after changing *ho* or *cnc* mRNA level in the brain is summarized in Table 3 (Supplementary Figure S6 and Supplementary Table S7). Significant differences in the total activity were mostly observed in flies with chronic modifications of *ho* expression in the brain. Only *elav* > *ho* flies showed identical activity patterns to their parental controls, while *repo* > *ho* flies were generally less active. In the case of *elav* > *hoRNAi* flies, they were typically more active. Whereas the total activity of *repo* > *hoRNAi* flies was not significantly different from the control groups. Flies with modifications in *cnc* expression in the brain showed changes in their locomotor activity compared to *ho* since we found significant differences not only in chronic experiments but also in those at specific developmental stages. In chronically treated *elav* > *cnc* flies, no significant difference was observed in total activity, while *repo* > *ho* flies exhibited a reduction in their total activity. In flies with chronic *cnc* silencing either in neurons or glia, the total activity was lower when compared to parental controls. When changes in *cnc* expression level were introduced at different developmental stages of *Drosophila*, we found higher total activity in larva-specific *cnc* overexpression in glia, larva-specific *cnc* silencing in either neurons or glia and adult-specific *cnc* silencing in glia.

**Table 3:** Summary of the effects of chronic changes of *ho* or *cnc* mRNA level in neurons or glia on locomotor activity pattern in *Drosophila* adults.

Genotype	Timing			
	Chronic	Larva-specific	Pupa-specific	Adult-specific
*elav > ho*	ns	ns	ns	ns
*elav > hoRNAi*	Higher	ns	ns	ns
*repo > ho*	Lower	ns	ns	ns
*repo > hoRNAi*	ns	ns	ns	ns
*elav > cnc*	ns	ns	ns	ns
*elav > cncRNAi*	Lower	Higher	ns	ns
*repo > cnc*	Lower	Higher	ns	ns
*repo > cncRNAi*	Lower	Higher	ns	Higher

The following remarks are described as higher for a significant increase in total activity, lower for a significant decrease in total activity, and ns for no significant difference in total activity.

### 3.3. Effects of changing *ho* or *cnc* expression in the brain at different developmental stages on sleep in adults

Sleep was also checked in adults with modifications of *ho* expression during their development. We found significant differences in sleep when *ho* expression was upregulated or downregulated in the brain at different developmental stages (Table 4; Supplementary Table S8).

**Table 4:** Summary of the effects of changing *ho* mRNA level in neurons or glia at specific developmental stages on sleeping pattern (daytime and nighttime sleep) of *Drosophila* adults.

Genotype	Timing			
	Chronic	Larva-specific	Pupa-specific	Adult-specific
*elav > ho*	↑ in daytime sleep	ns	ns	ns
*elav > hoRNAi*	↓ in daytime sleep	ns	ns	ns
*repo > ho*	↑ in daytime & nighttime sleep	↓ in daytime sleep	ns	↑ in daytime sleep
*repo > hoRNAi*	ns	ns	ns	↑ in daytime sleep
*elav > cnc*	↑ in daytime & nighttime sleep	ns	↑ in daytime sleep	ns
*elav > cncRNAi*	↑ in daytime & nighttime sleep	↓ in daytime & nighttime sleep	↓ in nighttime sleep	ns
*repo > cnc*	↑ in daytime & nighttime sleep	↓ in nighttime sleep	ns	↑ in daytime sleep
*repo > cncRNAi*	↑ in daytime & nighttime sleep	↓ in daytime & nighttime sleep	↑ in daytime sleep ↓ in nighttime sleep	ns

The following remarks are described as ↑ for a significant increase in sleep, ↓ for a significant decrease in sleep, and ns for no significant difference in daytime and nighttime sleep.

**Figure 6:**
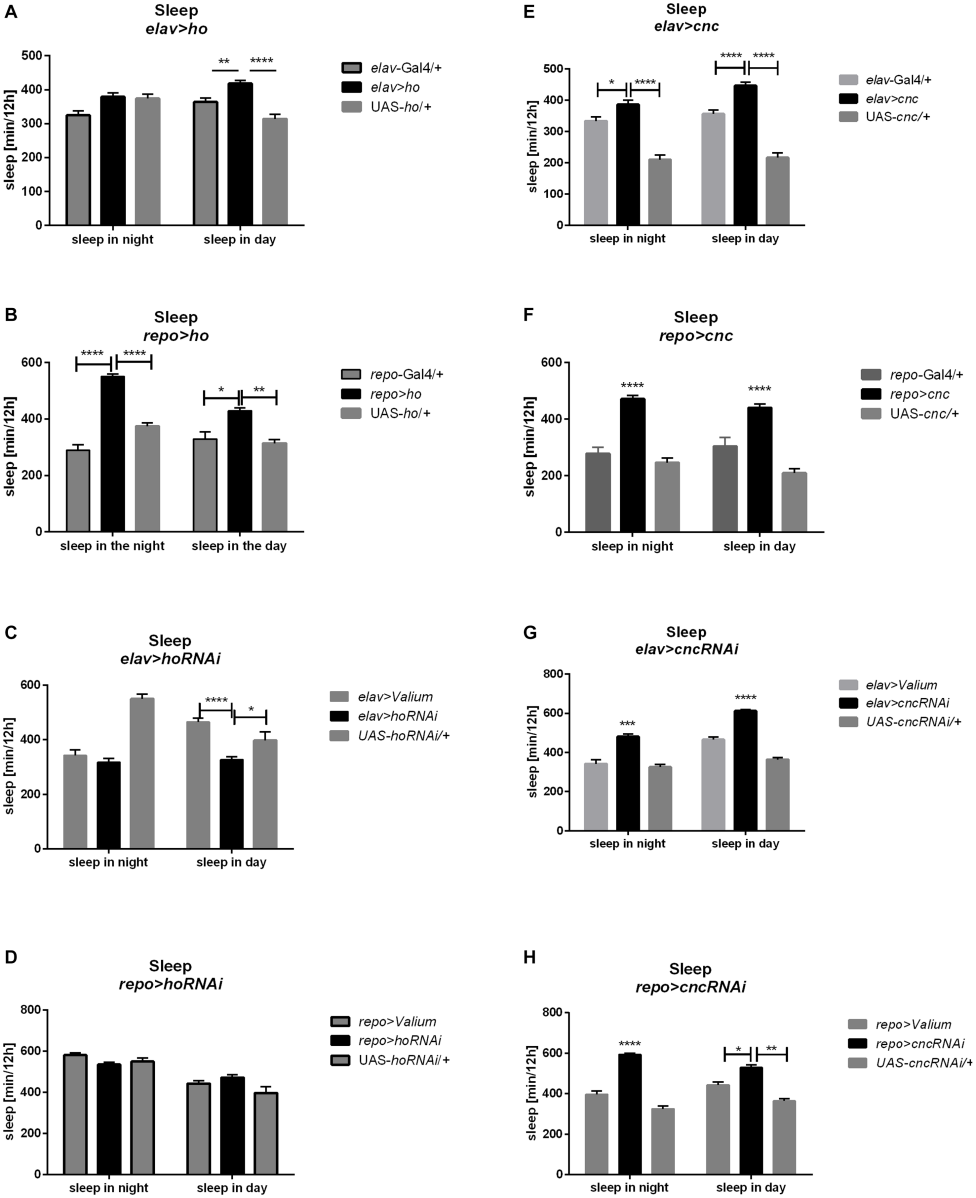
The sleep level of *Drosophila* adults is altered by chronic changes in *ho* **(A–D)** or *cnc* **(E–H)** mRNA level in the brain, specifically in neurons or glia. Data are expressed as means ± SE. Statistically significant differences between genotypes are indicated with *asterisks* (^*^p < 0.05, ^**^p < 0.01, ^***^p < 0.001, ^****^p < 0.0001).

When *ho* was chronically overexpressed in the brain, changes in sleep patterns were significant compared to their parental controls. In *elav* > *ho* flies, daytime sleep was higher while nighttime sleep was unaffected (Figure 6A and Supplementary Table S8). On the other hand, daytime and nighttime sleep increased in *repo* > *ho* adults (Figure 6B). Pan-neuronal *ho* overexpression at specific developmental stages did not produce any significant results in sleep patterns. On the contrary, different responses were observed after overexpressing *ho* in the glia at specific developmental stages. Nighttime sleep was not affected by expression changes made during the larval, pupal, or adult stages. But then, opposite effects were observed regarding daytime sleep when pan-glial *ho* overexpression was induced at the larval or adult stage. Daytime sleep was reduced in flies with larva-specific *ho* overexpression in the glia, while it was increased in flies treated during the adult stage. In pupa-specific *ho* overexpression in glia, the daytime sleep was not changed at all. The effects of *ho* silencing in the brain on sleep in adults were rather weak (Figures 6C,D). We only found significant differences in daytime sleep in chronically treated *elav* > *hoRNAi*, which was shorter, and in adult-specific *ho* silencing in glia which had longer daytime sleep.

The chronic overexpression of *cnc* in neurons or glia brough similar results in daytime and nighttime sleep, which were longer in comparison to their respective parental controls (Figures 6E,F). On the other hand, continuous silencing of *cnc* in neurons or glia showed similar effects, increasing daytime and nighttime sleep (Figures 6G,H). Activating *cnc* overexpression at specific developmental stages generated different effects. The sleeping pattern was changed only when pan-neuronal overexpression of *cnc* was carried out at the pupal stage and daytime sleep was increased in this case. For pan-glial overexpression of *cnc*, we only found significant differences in sleep after overexpression at the larval stage (with decreased nighttime sleep), and at the adult stage (increased daytime sleep). On the other hand, continuous silencing of *cnc* in neurons or glia brough similar effects because daytime and nighttime sleep increased in both cases. It was the opposite after larvae-specific silencing of *cnc* in neurons or glia since both daytime and nighttime sleep was decreased. Pupae-specific downregulation of *cnc* in the brain reduced nighttime sleep, however, when directed to glia, it also increased daytime sleep. The partial suppression of *cnc* in either neurons or glia during the adult stage did not bring any significant changes in the sleeping behavior of adult flies.

### 3.4. The mRNA level of *ho* and *cnc* at different developmental stages

**Figure 7:**
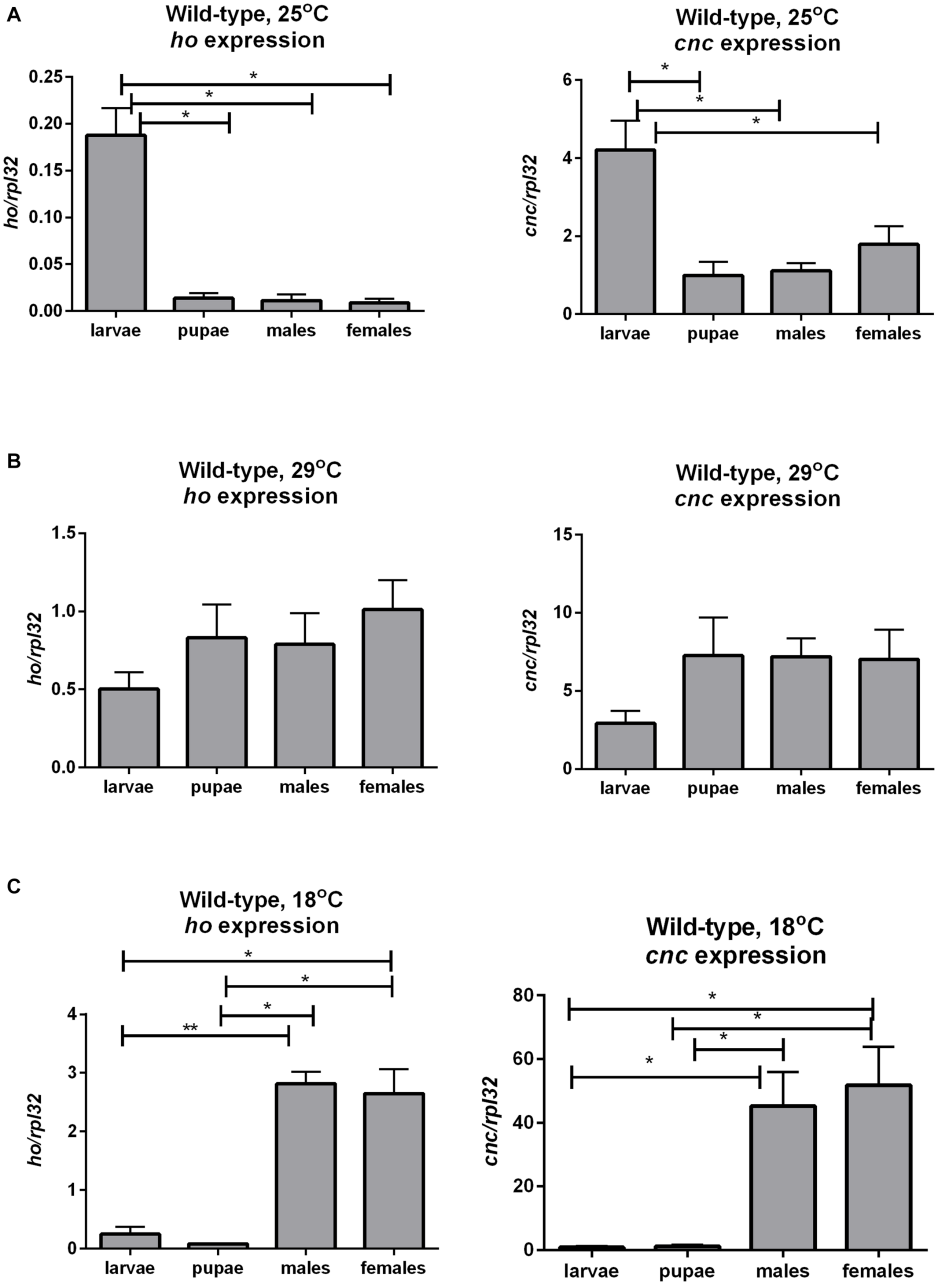
The mRNA level of *ho* and *cnc* in larvae, pupae, and in heads of adults (males and females) of wild-type (CS) flies changes with ambient temperature conditions: 25°C **(A)**, 29°C **(B)**, and 18°C **(C)**. Data are expressed as means ± SD. Statistically significant differences between groups are indicated with *asterisks* (^*^p < 0.05 and ^**^p < 0.01).

To have baseline data about the transcriptional regulation of *ho* and *cnc* during development in *Drosophila*, we examined *ho* and *cnc* mRNA levels at different life stages of wild-type flies. Both *ho* and *cnc* mRNA levels were similarly maintained at higher levels during the larval stage compared to the pupal and adult stages under optimal temperature conditions (Figure 7A and Supplementary Table S8). At 29°C, there were no significant changes in *ho* and *cnc* mRNA levels between CS larvae (whole body), pupae (whole body), and adults (head) (Figure 7B and Supplementary Table S9). At 18°C, both *ho* and *cnc* mRNA were significantly higher in adults (head) compared to larvae (whole body) and pupae (whole body) (Figure 7C and Supplementary Table S9).

**Figure 8:**
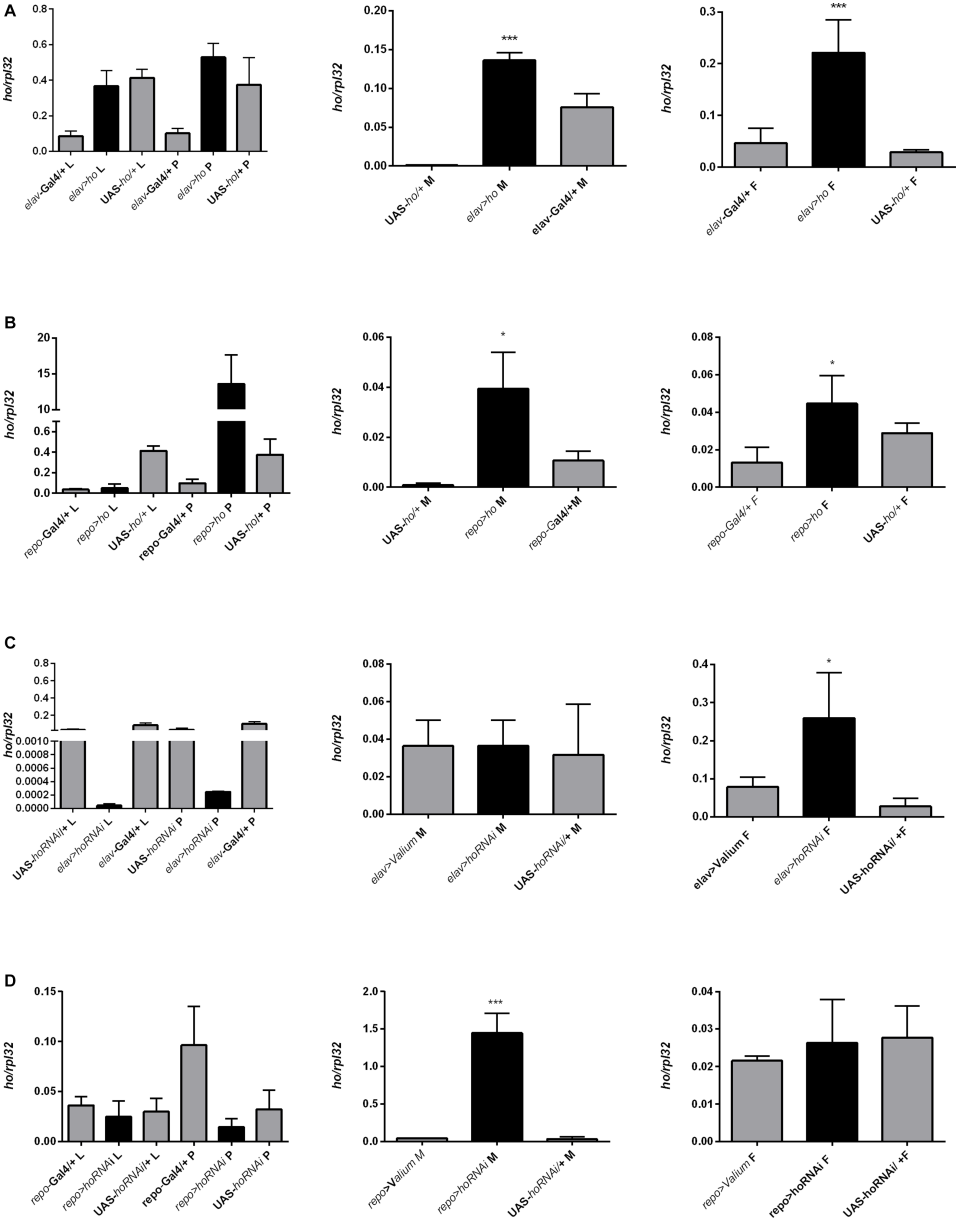
The *ho* expression pattern in larvae (L), pupae (P), and in heads of *Drosophila* adults (M for males, F for females) after chronic overexpression or silencing of *ho* in either neurons **(A,C)** or glia **(B,D)**. Data are shown as means ± SE. Statistically significant differences between genotypes are indicated with *asterisks* (^*^p < 0.05 and ^***^p < 0.001).

**Figure 9:**
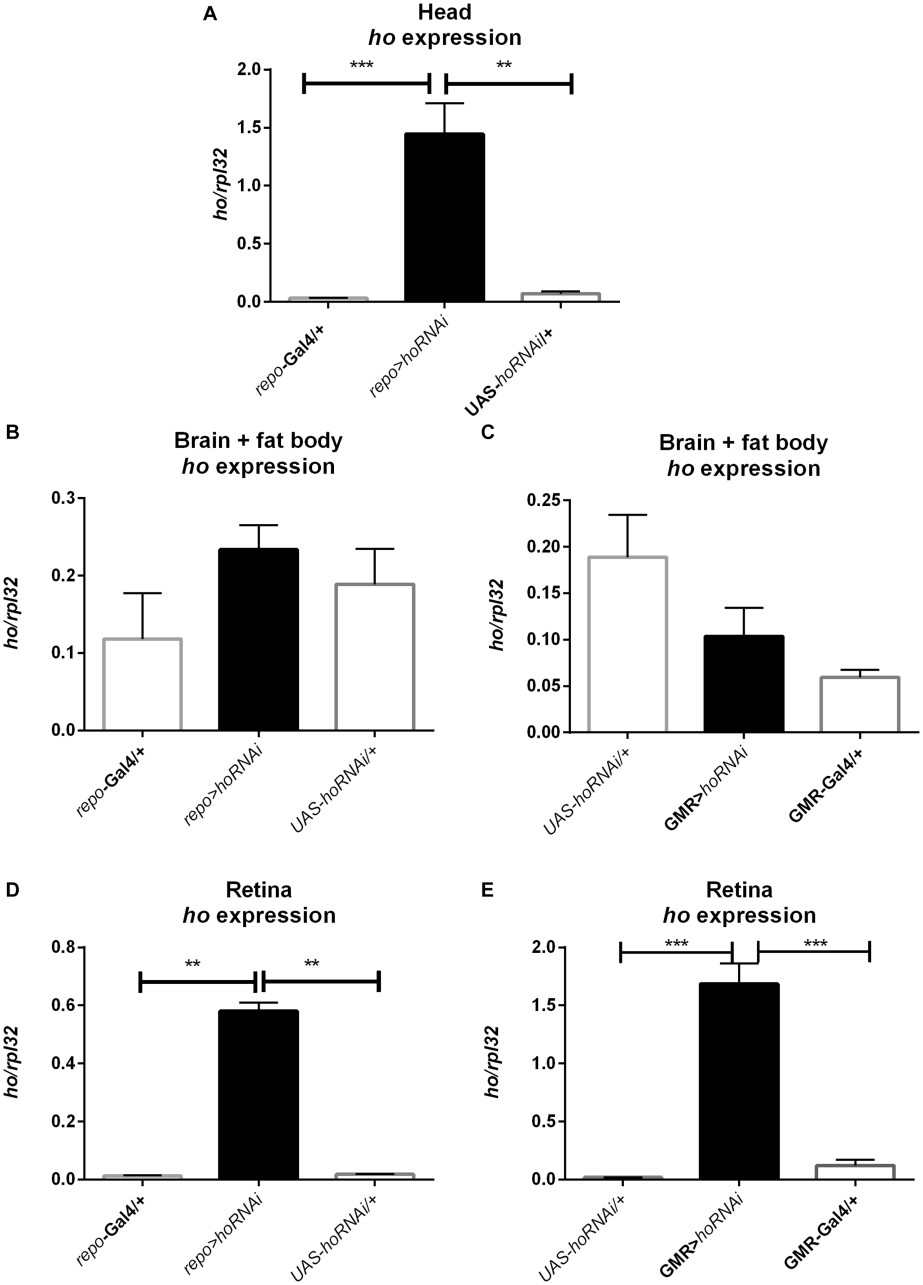
The increase of *ho* mRNA in the heads of adult flies with chronic *ho* silencing in glia **(A)** is not from the brain **(B,C)** but mostly from the retina **(D,E)**. Data are presented as means ± SE. Statistically significant differences between genotypes are indicated with *asterisks* (^**^p < 0.01 and ^***^p < 0.001).

The mRNA level of *ho* was also examined in larvae (whole body), pupae (whole body), and adults (heads of males and females, separately) after modifying its expression chronically or at specific developmental stages. Surprisingly, we did not find any significant differences in *ho* mRNA levels in larvae, pupae, and heads of adults after changing *ho* expression, specifically during the larval, pupal, or adult stage (Supplementary Table S8). Nevertheless, we found a trend of *ho* mRNA increase in larvae, pupae, or adults in flies with *ho* overexpression and unaffected or lesser *ho* mRNA in flies with RNAi. However, the differences were not statistically significant. We only observed significant changes in *ho* mRNA levels in male and female adults (heads) after chronic expression. The continuous *ho* overexpression in both neurons and glia induced significantly higher *ho* mRNA levels in the heads of males and females (Figures 8A,B and Supplementary Table S9). On the contrary, we observed significantly high *ho* mRNA levels in the heads of female *elav* > *hoRNAi* and male *repo* > *hoRNAi* flies (Figures 8C,D and Supplementary Table S9). We suspected compensatory effects after partially suppressing *ho* in neurons or glia. We found that the high expression of *ho* originated from the retina (Figure 9 and Supplementary Table S9). In the brain, *ho* mRNA level did not change. It seems that the compensatory effect occurs when the compound eye is fully developed as we did not find any changes in *ho* mRNA level in larvae or pupae. However, it is worth mentioning that we manipulated *ho* expression only in specific cell types, neurons or glia, and this change can be difficult to detect in whole head samples.

**Figure 10:**
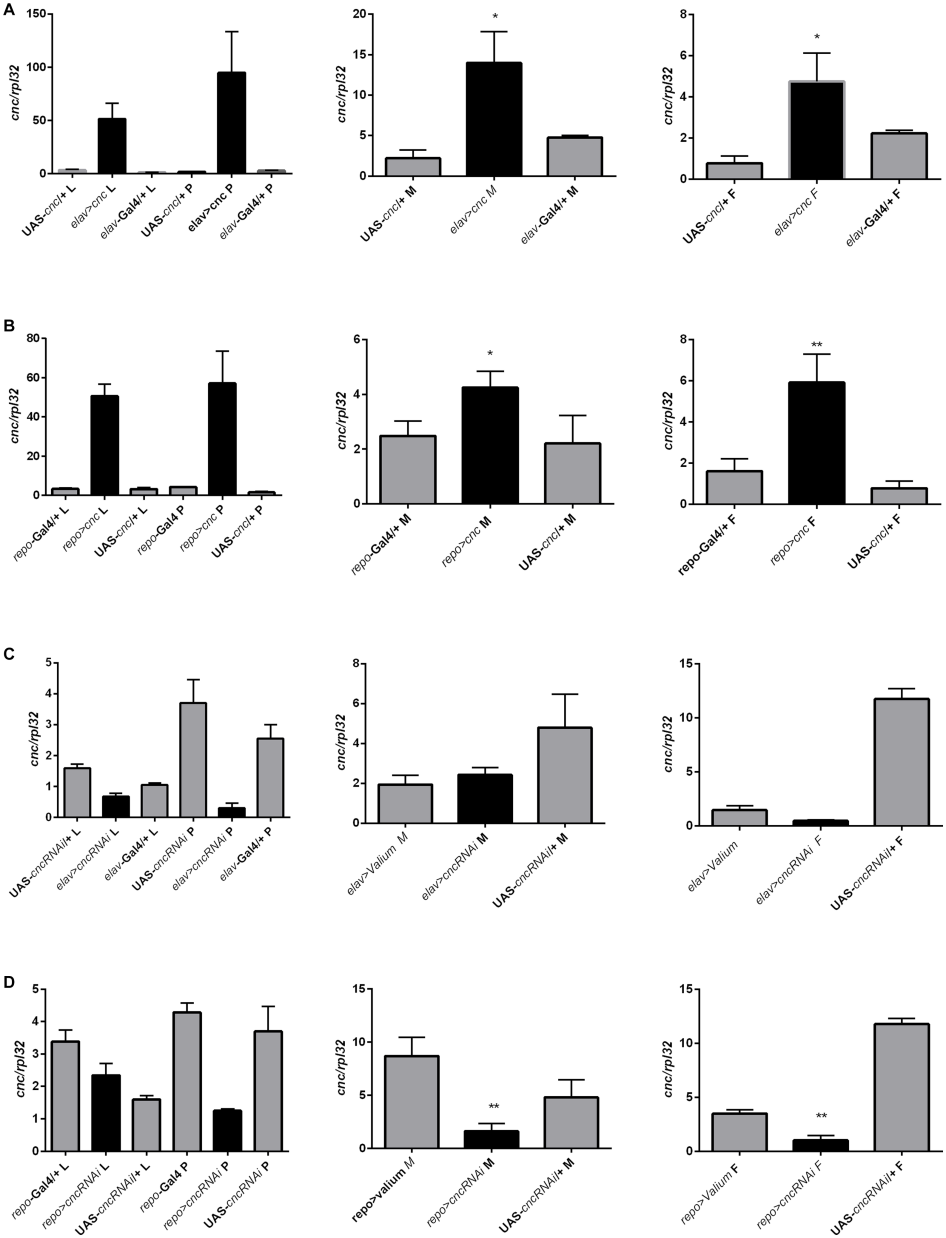
The *cnc* expression pattern in larvae (L), pupae (P), and heads of *Drosophila* adults (M for males, F for females) after chronic overexpression or silencing of *ho* in either neurons **(A,C)** or glia **(B,D)**. Data are expressed as means ± SE. Statistically significant differences between genotypes are indicated with *asterisks* (^*^p < 0.05 and ^***^p < 0.001).

We also quantified *cnc* mRNA levels in larvae (whole body), pupae (whole body), and adults (head) after the chronic modification of *cnc* expression in the brain, however, significant differences between experimental and control groups were only detected in adults. Chronic overexpression of *cnc* in neurons elevated *cnc* mRNA levels in both males and females (Figure 10A and Supplementary Table S9). In glia, *cnc* overexpression was only detected in females; however, we observed a similar trend in males (Figure 10B and Supplementary Table S9). The reduction of *cnc* transcript level was detected only in males and females after chronic *cnc* silencing in glia (Figure 10C and Supplementary Table S8). We found no significant differences when *cnc* was silenced in neurons (Figure 10D and Supplementary Table S9). Nonetheless, *cnc* mRNA levels were similar to controls.

## 4. Discussion

In the present study, we showed that disruption of the normal physiological level of heme oxygenase (HO) during development affects the adult life of *Drosophila melanogaster*.

First we found that the expression of *ho* and *cnc* varies between developmental stages and depends on temperature. At 25°C it was the highest in larvae and lower in pupae and adults. At 18°C, however, the expression level was reversed with the highest level in adults and at 29°C the mRNA level was similar during all developmental stages. This indicates that temperature exposure during rearing can affect experiments involving genetic manipulations. In addition, the TARGET system, which is commonly used in fly research, also needs to be used with a great care, since stage-specific genetic modification requires temperature changes between 18°C and 29°C, which are not optimal for flies, and can affect physiological processes in the adult life. Moreover, the temperature of 29°C drives expression of thermo-responsive miRNA, which affects gene expression and transposition (Chen et al., 2015; Fast and Rosenkrantz, 2018).

The effect of HO on adult longevity (survival and maximum lifespan) was more pronounced and consistent than on fitness (climbing ability and locomotor activity) and sleep of adult flies. We found that chronic modifications of *ho* expression (either upregulated or downregulated) in the brain results in higher survival compared with controls. It is possible that continuous overexpression or silencing of *ho* through development to adulthood affects many crucial processes dependent on HO level (Zhang et al., 2004; Cui et al., 2008; Ida et al., 2013; Klemz et al., 2017; Damulewicz et al., 2017a,b, 2019; Abaquita et al., 2021, 2023), including the circadian clock (Mandilaras and Missirlis, 2012; Klemz et al., 2017; Damulewicz et al., 2017a, 2019). The clock disruption can alter circadian rhythms in many physiological and behavioral processes (Ceriani et al., 2002). It may also explain why fitness was significantly changed in flies with chronic *ho* overexpression or silencing in the brain compared with changes that were larva, pupa-, and adult-specific.

When changes in *ho* expression were induced at specific developmental stages, we observed an opposite effect on adult survival. The survival was mostly decreased compared to their respective controls. This indicates that HO in the brain is tightly regulated during development to adulthood since changing *ho* expression at different developmental stages can disrupt the longevity of adults. There are, however, exemption, because the survival did not change after increasing *ho* expression in neurons at the larval stage and decreasing it in neurons at the pupal stage. It is important to highlight these findings because, for instance, inducing *ho* expression in larvae does not affect longevity, fitness, and sleep of adults. This suggests that a high physiological level of HO is important for larvae. After checking the *ho* mRNA level at different developmental stages, we found that the *ho* gene is highly expressed in larvae under physiological conditions. It has also been reported that *ho* mRNA level is high in the third-instar larvae (L3, specifically at puff stage 1–9), using a high-throughput expression analysis (Brown et al., 2014) which may be associated with the physiological demand for the synthesis of hormones (i.e., juvenile hormone and ecdysteroids) regulating molting and metamorphosis (Massie et al., 1985; Llorens et al., 2015). The synthesis of both ecdysone and juvenile hormone requires functional cytochrome P450 which contains heme as a cofactor. The high expression of *ho* in larvae is probably related to its cytoprotective and antioxidative functions to prevent free heme deposition (Linzke et al., 2014). HO might have the same functions in pupae (Brown et al., 2014), however, partial suppression of *ho* is not critical according to our results. Active heme metabolism is essential during the larval stage as HO deficiency in L3 leads to the increase of heme and iron pools (Cui et al., 2008). The same authors also reported that 95% knockdown of the *ho* gene is lethal at the larval stage since a few viable L3 were observed and they did not survive metamorphosis.

All available data regarding the level of *ho* and *cnc* expression at specific stages of development are not consistent, however. According to the modENCODE Anatomy RNA-Seq, in L3 wandering larvae *ho* is at its high level in the digestive system and fat body and it is moderate in the nervous system, while FlyAtlas Anatomical Expression Data indicate extremely high *ho* mRNA level in the fat body, but not in the digestive system. We decided to use whole body of larvae and pupae to analyse the whole nervous system, including the peripheral one. Because of that mRNA levels of *ho* and *cnc,* which we measured, did not show statistically significant changes between experimental and control groups. However, we used qPCR to quantify mRNA, which provides precise data on gene expression. Because of that we could focus on one selected gene, since primers were designed specifically for the gene of interest and reaction specificity was checked with melt curve analysis. The obtained results showed a growing effect of silencing/overexpression of *ho* and *cnc* from larvae to pupae, and in adults statistically significant changes were detected. Moreover, our results confirmed the modENCODE Temporal Expression Data that in larvae the mRNA level of *ho* is higher than in pupae and adults, and almost the same in males and females.

Similarly, mammalian HO-1 (homolog of HO in *Drosophila*) is highly induced during embryonic development supporting the survival of the embryo (Ihara et al., 1998; Watanabe et al., 2004; Zhao et al., 2009; Zenclussen et al., 2015; Loboda et al., 2016). Deletion of HO-1 in mice leads to high prenatal lethality and surviving adults with HO-1 deficiency are smaller, they breed poorly and are less active. They also have several abnormalities leading to high mortality (Poss and Tonegawa, 1997a,b; Kovtunovych et al., 2010). In contrast, HO-1 overexpression improves embryonic development (Zenclussen et al., 2006, 2015). It has been suggested that dysfunction of HO can affect the release of its metabolite CO (Zenclussen et al., 2011, 2015) which acts against oxidative stress (Linzke et al., 2014) and probably maintains homeostasis of glucose (Klemz et al., 2017).

We also observed sex- and age-specific differences in the longevity and fitness of *Drosophila* adults. Regarding maximum lifespan, females had usually longer lifespans compared to males. It was previously shown that *Drosophila* females live longer, and their aging is delayed because of higher antioxidative enzyme expression in old age (Deepashree et al., 2017; Niveditha et al., 2017). In addition, there are sex-specific differences in stress response and stress adaptation (see Tower et al., 2020), including the regulation of the growth and cell signaling pathways, organ homeostasis and metabolism (Magwere et al., 2004; Millington and Rideout, 2018), and the HO system (Barañano and Snyder, 2001; Han E. et al., 2005; Han Y. et al., 2005; Ren et al., 2021). Another evidence is that the survival pattern of females is less affected than males, specifically when *ho* expression is reduced in neurons at the larval or adult stage and induced in glia at the pupal stage. One exemption was when *ho* was silenced in glia at the pupal stage, and this affected only females but not males. We also found that increasing *ho* expression in glia decreases climbing ability in 30 days-old females while decreasing *ho* expression adversely affects the climbing performance of 30 days-old males.

It seems that the influence of HO in the brain on the survival of aging flies depends on which cells in the nervous system are affected. In our chronic experiments, neuronal *ho* overexpression resulted in a higher percentage of survival of young flies while neuronal *ho* silencing promoted better survival of aging flies. Glial *ho* expression changes were critical for aging flies only. It means that HO in neurons and glial cells has distinct functions during development until adulthood which has already been reported in mammals (see Schipper et al., 1995, 2006; Song et al., 2007; Schipper et al., 2009; Ohnishi et al., 2010; Tronel et al., 2013; Barone et al., 2014; Mena et al., 2015; Chiang et al., 2018; Nitti et al., 2018; Waza et al., 2018; Schipper et al., 2019; Luu Hoang et al., 2021; Wu and Hsieh, 2022; Yang and Wang, 2022).

Sleep is very sensitive to changes in an organism’s homeostasis and is regulated by the circadian clock. Here, the sleep pattern was changed in adults after *ho* overexpression or silencing in the brain. In our chronic expression experiments, different sleep patterns were observed depending on the level of *ho* expression in neurons and glia. Overexpression of *ho* in the glia increased both daytime and nighttime sleep, which did not change when *ho* expression in the glia was downregulated. Flies with *ho* overexpression in neurons had longer daytime sleep compared to controls, but when *ho* was silenced, daytime sleep was reduced. When *ho* expression was changed at specific developmental stages, the sleep pattern was either unaffected or results were inconsistent.

The effects observed in adult flies after *ho* overexpression or silencing in either neurons or glia at specific developmental stages, were not always clear and consistent with those after chronic treatments. It might be due to high and low temperatures used to induce and suppress, respectively *ho* expression. We found differences in the relative abundance and expression pattern of *ho* mRNA after exposing immature and adult *Drosophila* to low or high temperatures than to the optimal one. Under heat stress aging and metabolism are faster, while in lower temperatures, both processes are slow down (Mołoń et al., 2020). For that reason, HO activity can also change under different temperature conditions.

In addition, the high-throughput expression analyses of the Fly Cell Atlas project (Li et al., 2022) revealed that basal *ho* expression in neurons is maintained at low to moderate levels. Dysregulation of heme metabolism may be stressful for neurons and glia. The appropriate level of HO in the brain is so important that there are compensatory mechanisms that help to maintain its expression. When *ho* was partially silenced in neurons or glia, we surprisingly observed overexpression of *ho* in heads. More detailed analysis showed that the retina produces high amount of HO that compensates the lack of this enzyme in other cells. We did not identify the cell type, which is responsible for this process, as silencing in both glia and photoreceptors showed increased *ho* expression in the retina. Additional evidence suggesting that this mechanism depends on the retina comes from developmental studies—we did not observe this compensatory effect in larvae, which do not have compound eyes. It started to progress in pupae and then it was strongly manifested in adults. This phenomenon is very interesting as in most cases genetic compensation is observed only in mutants but not after knockdowns and it occurs through the overexpression of different genes to avoid the negative effect of mutation (El-Brolosy and Stainier, 2017).

Interestingly, disrupting the *cnc* expression, a gene encoding one of the active isoforms of *Drosophila* CNC in the brain also affected adult flies, depending on the timing, expression level, cell types, sex, and age of adults. However, the obtained results were different than after manipulating *ho* expression. We expected that CNC-dependent genes like *ho,* should have similar effects as after changing *cnc* expression (Loboda et al., 2016). CNC is a multifunctional transcription factor that regulates growth and development, proteasome stability and detoxification (Loboda et al., 2016). Its function begins during oogenesis (Guichet et al., 2001). We found that *cnc* is highly expressed in larvae, but its transcript level is comparably lower in pupae and adults. The CNC transcription factors play an important role in aging (Loboda et al., 2016), which can explain the differences in adult life parameters between *cnc* and *ho* after modifying their expression in the brain.

In conclusion, adult life depends on adequate HO levels in the brain during development. Our findings indicate that the appropriate level of HO at the early stage of life can prevent health problems during aging. It seems to be similar in flies and mammals, because multiple cellular processes are affected by heme metabolism dysfunction, leading to differences in longevity, fitness, and sleep under various conditions.

## Data availability statement

The datasets presented in this study can be found in online repositories. The names of the repository/repositories and accession number(s) can be found at: https://doi.org/10.57903/UJ/UD75IP, UD75IP.

## Ethics statement

The manuscript presents research on animals that do not require ethical approval for their study.

## Author contributions

BB and MD carried out all experiments. MD performed all data analyses and prepared figures. TA summarized the results and wrote the first draft of the manuscript. EP finalized the concept of the study, validated all results, interpreted the data, and together with MD, prepared the final version of the manuscript. All authors contributed to the article and approved the submitted version.

## Footnotes

^1^ http://www.R-project.org
