# Supplementary material for: Changes in heme oxygenase level during development affect the adult life of Drosophila melanogaster
Source: Front Cell Neurosci. 2023 Oct 9;17:1239101. doi: 10.3389/fncel.2023.1239101 (PMC10591093; doi:10.3389/fncel.2023.1239101)
Supplement: Supplementary file 1 [file Table_1.DOCX]

**Supplementary Table 1**. Detailed statistics for survival (for Figures 1-4).

| **GENOTYPE** | **TIMING** | **S** | **HEALTH SPAN** | **TRANSITION PHASE** | **SENESCENCE PHASE** | **MAXIMUM LIFESPAN (ML)** | **ML (CTRs)** | ***P* (ML)** | **MEDIAN** | **MEDIAN (CTRs)** | **MEAN** | **MEAN (CTRs)** | ***N*** | ***N* (CTRs)** |
| --- | --- | --- | --- | --- | --- | --- | --- | --- | --- | --- | --- | --- | --- | --- |
| *elav>ho* | Chronic | M | ***0.006***  ***0.001*** | ***<0.001***  ***0.003*** | *0.1*  *0.07* | 75 | 77  69 | *0.8*  *0.005* | 49 | 47.5  47 | 46.4 | 42.3  35.9 | 92 | 84  84 |
|  |  | F | ***<0.001***  ***<0.001*** | *<0.001*  *0.6* | ***<0.001***  ***0.02*** | 93 | 81  96 | *<0.001*  *0.4* | 77 | 34.5  72 | 74.5 | 40.1  54.5 | 92 | 86  84 |
|  | Larva-specific | M | *0.1*  *0.06* | *0.4*  *0.7* | *0.5*  *0.001* | 176 | 172  155 | *0.1*  *0.04* | 112 | 125  111 | 104.5 | 117.3  107.5 | 95 | 89  89 |
|  |  | F | *0.4*  *0.02* | *0.08*  *0.6* | *0.1*  *0.06* | 176 | 186  185 | *0.4*  *0.01* | 130 | 128  142 | 116.3 | 119.5  135.3 | 99 | 97  99 |
|  | Pupa-specific | M | *<0.001*  *0.05* | *0.01*  *0.8* | *0.9*  *0.5* | 166 | 161  167 | *0.005*  *0.2* | 92 | 117  100 | 90.2 | 113.9  101.4 | 107 | 108  71 |
|  |  | F | ***0.03***  ***0.01*** | ***<0.001***  ***<0.001*** | *<0.001*  *0.7* | 181 | 160  185 | *0.9*  *0.003* | 96 | 131  152 | 102.3 | 127.6  131.6 | 101 | 88  87 |
|  | Adult-specific | M | ***<0.001***  ***0.006*** | *0.002*  *0.8* | *0.7*  *0.8* | 48 | 49  51 | *<0.001*  *0.08* | 24.5 | 38  29 | 25.1 | 37.7  29.3 | 90 | 90  89 |
|  |  | F | *<0.001*  *0.2* | *<0.001*  *0.8* | *0.8*  *0.9* | 49 | 53  52 | *<0.001*  *0.5* | 25 | 42  30 | 28.6 | 38.2  30.9 | 90 | 90  90 |
| *elav>hoRNAi* | Chronic | M | *<0.001*  *0.003* | *0.5*  *0.7* | ***0.03***  ***<0.001*** | 82 | 81  69 | ***<0.001***  ***0.005*** | 47 | 28.5  35 | 48.5 | 31.7  40.3 | 80 | 94  91 |
|  |  | F | ***<0.001***  ***<0.001*** | *0.08*  *<0.001* | *0.07* | 85 | 82  50 | ***<0.001***  ***<0.001*** | 66 | 18  33.5 | 63.8 | 34  33.6 | 90 | 72  96 |
|  | Larva-specific | M | *<0.001*  *0.8* | ***<0.001***  ***<0.001*** | *0.3*  *<0.001* | 166 | 154  186 | ***0.003***  ***<0.001*** | 91 | 105  125 | 89.1 | 88  121.5 | 87 | 89  106 |
|  |  | F | *0.007*  *0.3* | *0.08*  *0.7* | *<0.001*  *0.3* | 194 | 177  193 | *<0.001*  *0.5* | 147 | 112  156 | 134.6 | 104.5  133.3 | 91 | 101  135 |
|  | Pupa-specific | M | *0.9*  *0.03* | *0.09*  *<0.001* | *0.7*  *<0.001* | 124 | 140  182 | *0.08*  *<0.001* | 75 | 82.5  129 | 75.9 | 80.5  112 | 66 | 92  63 |
|  |  | F | *0.6*  *<0.001* | *0.001*  *0.2* | *0.2*  *<0.001* | 177 | 162  183 | *0.006*  *<0.001* | 126 | 119  152.5 | 127.8 | 114.2  130.2 | 92 | 91  50 |
|  | Adult-specific | M | *0.08*  *0.2* | ***<0.001***  ***<0.001*** | *0.2*  *0.02* | 48 | 52  45 | *<0.001*  *<0.001* | 38 | 35.5  37 | 39.4 | 34.8  35.1 | 90 | 90  90 |
|  |  | F | *0.7*  *0.3* | *0.3*  *0.009* | *<0.001*  *0.7* | 52 | 53  51 | *<0.001*  *0.9* | 44 | 48  45 | 42.3 | 45.1  44.2 | 90 | 89  90 |
| *repo>ho* | Chronic | M | ***<0.001***  ***<0.001*** | ***0.04***  ***0.003*** | *0.8*  *0.9* | 73 | 72  71 | ***<0.001***  ***<0.001*** | 54.5 | 14  47 | 49.6 | 20.5  35.8 | 88 | 74  85 |
|  |  | F | ***<0.001***  ***<0.001*** | *<0.001*  *0.8* | *0.2*  *0.2* | 97 | 72  96 | ***<0.001***  ***<0.001*** | 78 | 17  72 | 76 | 22.9  54.6 | 92 | 104  92 |
|  | Larva-specific | M | ***<0.001***  ***<0.001*** | ***0.004***  ***0.002*** | ***0.002***  ***<0.001*** | 134 | 146  142 | ***<0.001***  ***<0.001*** | 42 | 108  100 | 55.2 | 97.8  86.5 | 90 | 89  90 |
|  |  | F | ***<0.001***  ***<0.001*** | ***<0.001***  ***<0.001*** | *0.05*  *0.03* | 153 | 151  151 | ***<0.001***  ***<0.001*** | 39 | 114  84.5 | 49.6 | 101.5  80 | 90 | 84  90 |
|  | Pupa-specific | M | ***<0.001***  ***<0.001*** | ***<0.001***  ***<0.001*** |  | 53 | 142  128 | ***<0.001***  ***<0.001*** | 35 | 108  90 | 35.4 | 105.3  79.3 | 30 | 90  85 |
|  |  | F | *0.04*  *0.5* | *0.6*  *0.5* | *<0.001*  *0.9* | 153 | 142  161 | *0.006*  *0.7* | 107.5 | 107  120 | 97.2 | 102.7  104.4 | 90 | 90  78 |
|  | Adult-specific | M | *<0.001*  *0.8* | *0.06*  *0.2* | *0.08*  *0.6* | 49 | 42  48 | *<0.001*  *0.8* | 38 | 12  41 | 35.1 | 20.6  36 | 120 | 91  150 |
|  |  | F | *<0.001*  *0.7* | *0.1*  *0.7* | ***0.007***  ***0.01*** | 52 | 49  51 | *<0.001*  *0.1* | 39 | 13  38 | 32.3 | 19.1  32.9 | 150 | 199  131 |
| *repo>hoRNAi* | Chronic | M | *0.4*  *0.003* | ***<0.001***  ***0.001*** | ***0.006***  ***<0.001*** | 82 | 69  69 | ***<0.001***  ***<0.001*** | 57.5 | 46  35 | 51.5 | 42.7  40.3 | 96 | 93  91 |
|  |  | F | *0.2*  *<0.001* | ***<0.001***  ***<0.001*** | *0.03* | 87 | 86  42 | *0.1*  *<0.001* | 73 | 60  31 | 70.7 | 59  31.8 | 92 | 84  84 |
|  | Larva-specific | M | ***<0.001***  ***<0.001*** | *0.1*  *0.6* | ***<0.001***  ***0.006*** | 149 | 129  138 | *0.5*  *0.2* | 81.5 | 94  100 | 73.2 | 85.2  99.2 | 90 | 90  93 |
|  |  | F | ***<0.001***  ***<0.001*** | *0.4*  *0.4* | ***<0.001***  ***<0.001*** | 166 | 140  151 | *0.9*  *0.9* | 71.5 | 119.5  106 | 80.7 | 104.6  106.7 | 90 | 74  87 |
|  | Pupa-specific | M | *0.1*  *0.6* | *0.5*  *0.002* | *0.4*  *<0.001* | 122 | 115  154 | *0.6*  *<0.001* | 84.5 | 82  91 | 77.9 | 80.6  93.7 | 90 | 90  77 |
|  |  | F | *0.3*  *<0.001* | ***0.01***  ***<0.001*** | *<0.001*  *0.07* | 144 | 161  156 | ***<0.001***  ***<0.001*** | 92 | 110  119 | 85.4 | 103.4  115.7 | 84 | 69  95 |
|  | Adult-specific | M | ***<0.001***  ***<0.001*** | *0.2*  *<0.001* | ***0.04***  ***<0.001*** | 38 | 43  45 | ***<0.001***  ***<0.001*** | 10.5 | 27  38 | 13.3 | 26.7  32.2 | 150 | 120  90 |
|  |  | F | ***<0.001***  ***<0.001*** | *0.07*  *0.2* | ***0.005***  ***0.01*** | 42 | 51  49 | ***<0.001***  ***<0.001*** | 13 | 37  35.5 | 16.2 | 32.6  33.3 | 180 | 44  142 |
| *elav>cnc* | Chronic | M | ***<0.001***  ***<0.001*** | *0.09*  *<0.001* | *0.02*  *0.05* | 76 | 73  71 | ***<0.001***  ***<0.001*** | 60 | 46  44 | 55.9 | 41.6  41.5 | 87 | 84  88 |
|  |  | F | *<0.001*  *0.07* | ***0.02***  ***<0.001*** | *0.02*  *<0.001* | 86 | 81  76 | ***<0.001***  ***<0.001*** | **64** | 28  54 | 54.5 | 38.5  48.6 | 77 | 82  90 |
|  | Larvae-specific | M | *0.2*  *0.4* | ***<0.001***  ***<0.001*** | ***0.03***  ***0.02*** | 169 | 172  163 | *<0.001*  *<0.001* | 79 | 125  126.5 | 84.9 | 117.3  107.2 | 71 | 89  96 |
|  |  | F | *0.8*  *0.5* | *0.6*  *0.2* | *0.02*  *0.7* | 173 | 186  170 | *0.03*  *0.9* | 113.5 | 128  131 | 111.4 | 119.5  114.1 | 106 | 97  90 |
|  | Pupae-specific | M | *0.4*  *0.1* | *0.7*  *<0.001* | *0.01*  *0.6* | 177 | 161  175 | *0.1*  *0.07* | 111.5 | 117  140 | 112.9 | 113.9  136.6 | 82 | 108  84 |
|  |  | F | *0.4*  *0.07* | ***0.001***  ***0.003*** | *0.1*  *0.5* | 177 | 160  172 | *0.7*  *0.2* | 122 | 131  133 | 115.5 | 127.6  129.9 | 66 | 88  96 |
|  | Adults-specific | M | *0.3*  *0.3* | *0.08*  *0.08* | ***0.002***  ***<0.001*** | 44 | 49  52 | *0.3*  *<0.001* | 40.5 | 38  44 | 38.5 | 37.7  43.3 | 90 | 90  90 |
|  |  | F | *1.0*  *<0.001* | *0.02*  *0.06* | *0.3*  *0.006* | 52 | 53  48 | *0.8*  *<0.001* | 42 | 42  38 | 39 | 38.2  32.4 | 90 | 90  91 |
| *elav>cncRNAi* | Chronic | M | ***<0.001***  ***<0.001*** | *0.05*  *<0.001* | *0.05* | 77 | 81  52 | *0.002*  *<0.001* | 45 | 38  14 | 43.6 | 32.3  17.9 | 89 | 94  96 |
|  |  | F | ***<0.001***  ***<0.001*** | ***0.03***  ***<0.001*** | ***<0.001*** | 93 | 82  49 | ***<0.001***  ***<0.001*** | 75 | 13  14 | 67.2 | 27.9  17.1 | 77 | 99  52 |
|  | Larvae-specific | M | *<0.001*  *0.2* | *0.07*  *0.2* | *0.8*  *0.8* | 158 | 154  148 | ***<0.001***  ***<0.001*** | 25 | 105  37 | 45.4 | 88  50.2 | 65 | 89  90 |
|  |  | F | *<0.001*  *0.3* | *<0.001*  *0.8* | ***<0.001***  ***<0.001*** | 123 | 177  141 | ***<0.001***  ***0.004*** | 29 | 112  43 | 51.9 | 104.5  56.5 | 91 | 101  96 |
|  | Pupae-specific | M | *<0.001*  *<0.001* | *<0.001*  *0.002* | *0.1*  *0.2* | 113 | 140  138 | *<0.001*  *0.6* | 71 | 82.5  41 | 63.7 | 80.5  52.3 | 93 | 92  96 |
|  |  | F | *<0.001*  *0.02* | *<0.001*  *<0.001* | *<0.001* | 127 | 162  100 | *<0.001*  *<0.001* | 82.5 | 119  61 | 74 | 114.2  54 | 92 | 91  85 |
|  | Adults-specific | M | *0.002*  *0.4* | ***<0.001***  ***<0.001*** | *0.002*  *0.2* | 44 | 52  44 | *<0.001*  *<0.001* | 29 | 35.5  35 | 26.6 | 34.8  31.6 | 90 | 90  90 |
|  |  | F | *<0.001*  *0.2* | *<0.001*  *0.2* | ***<0.001***  ***0.004*** | 44 | 47  53 | ***0.003***  ***<0.001*** | 29 | 32  48 | 27.8 | 31.3  45.1 | 90 | 90  89 |
| *repo>cnc* | Chronic | M | ***<0.001***  ***<0.001*** | ***<0.001***  ***<0.001*** | ***0.01***  ***<0.001*** | 82 | 73  71 | ***<0.001***  ***<0.001*** | 72 | 14.5  44 | 63.1 | 21.5  41.8 | 95 | 76  88 |
|  |  | F | ***<0.001***  ***0.01*** | ***<0.001***  ***0.02*** | *0.1*  *<0.001* | 95 | 72  76 | ***<0.001***  ***<0.001*** | 69 | 17  54 | 64 | 23.9  48.5 | 93 | 107  88 |
|  | Larvae-specific | M | *0.005*  *0.4* | *0.9*  *0.5* | *0.1*  *0.9* | 174 | 146  147 | *0.03*  *0.7* | 113 | 108  121 | 112.1 | 97.8  113.2 | 80 | 89  85 |
|  |  | F | *0.5*  *<0.001* | *0.6*  *0.01* | *0.7*  *0.7* | 151 | 151  151 | *0.8*  *0.03* | 112 | 114  124 | 98.7 | 101.5  120.8 | 90 | 84  90 |
|  | Pupae-specific | M | ***<0.001***  ***<0.001*** | ***<0.001***  ***<0.001*** | ***<0.001***  ***<0.001*** | 126 | 142  161 | ***<0.001***  ***<0.001*** | 85 | 108  115 | 77.4 | 105.3  112.9 | 90 | 90  90 |
|  |  | F | ***0.03***  ***0.04*** | ***<0.001***  ***<0.001*** | ***<0.001***  ***<0.001*** | 121 | 142  161 | ***<0.001***  ***<0.001*** | 97 | 107  119 | 81.2 | 102.7  115.7 | 81 | 90  90 |
|  | Adults-specific | M | *0.4*  *0.7* | ***<0.001***  ***<0.001*** |  | 32 | 42  43 | ***<0.001***  ***<0.001*** | 20 | 12  17 | 17.8 | 20.6  23.2 | 150 | 91  90 |
|  |  | F | *<0.001*  *0.01* | *<0.001*  *<0.001* | *0.04*  *0.3* | 48 | 49  49 | *0.3*  *<0.001* | 23 | 13  39 | 22.4 | 19.1  31.6 | 111 | 199  120 |
| *repo>cncRNAi* | Chronic | M | *0.2*  *<0.001* | *0.07*  *<0.001* | *0.1* | 74 | 83  52 | *0.1*  *<0.001* | 51 | 46  14 | 50 | 44.5  17.9 | 61 | 96  96 |
|  |  | F | *0.5*  *<0.001* | ***<0.001***  ***0.001*** | ***<0.001*** | 74 | 86  49 | *<0.001*  *<0.001* | 51 | 59.5  14 | 50 | 57.8  17.1 | 61 | 78  52 |
|  | Larvae-specific | M | ***<0.001***  ***<0.001*** | *0.02*  *0.2* | *0.1*  *0.009* | 117 | 129  125 | ***<0.001***  ***<0.001*** | 56 | 94  91 | 63.2 | 85.2  87.1 | 90 | 90  92 |
|  |  | F | *<0.001*  *0.1* | *<0.001*  *0.8* | ***<0.001***  ***0.02*** | 124 | 140  142 | *<0.001*  *0.04* | 85 | 119.5  92 | 74.2 | 104.6  81.1 | 90 | 74  98 |
|  | Pupae-specific | M | *0.002*  *0.4* | ***0.04***  ***<0.001*** | ***0.001***  ***<0.001*** | 106 | 115  126 | ***<0.001***  ***<0.001*** | 71 | 82  91 | 65.3 | 80.6  81.7 | 90 | 90  82 |
|  |  | F | *0.01*  *0.1* | *<0.001*  *0.8* | *<0.001*  *0.07* | 109 | 161  106 | *<0.001*  *0.1* | 67 | 110  64.5 | 63.8 | 103.4  59.6 | 90 | 69  90 |
|  | Adults-specific | M | *0.1*  *0.008* | ***<0.001***  ***<0.001*** | ***0.002***  ***0.002*** | 49 | 43  43 | ***<0.001***  ***0.004*** | 24 | 27  17 | 21.6 | 26.7  23.2 | 120 | 120  90 |
|  |  | F | *0.02*  *0.4* | *<0.001*  *0.3* | ***<0.001***  ***0.003*** | 44 | 49  45 | ***<0.001***  ***0.008*** | 29 | 35.5  28 | 26.3 | 33.3  28.4 | 120 | 142  30 |
