# Supplementary material for: Changes in heme oxygenase level during development affect the adult life of Drosophila melanogaster
Source: Front Cell Neurosci. 2023 Oct 9;17:1239101. doi: 10.3389/fncel.2023.1239101 (PMC10591093; doi:10.3389/fncel.2023.1239101)
Supplement: Supplementary file 2 [file Table_2.DOCX]

**Suplementary Table 5**. Detailed statistics for climbing assay in flies with modification of *ho* or *cnc* expression in males (M) and females (F). Experiments performed at 7-, 14-, 30-days old flies. Dunnett’s multiple comparison, experimental flies compared with parental controls (Gal4 and UAS). Three repetitions for every group, at least 20 flies per group.

| **Chronic** | **Gal4**  **p-value** | **UAS**  **p-value** | **Gal4**  **p-value** | **UAS**  **p-value** | **Gal4**  **p-value** | **UAS**  **p-value** |
| --- | --- | --- | --- | --- | --- | --- |
|  | **7 d** | | **14 d** | | **30 d** | |
| *repo>hoRNAi* M | 0.4402 | 0.6745 | 0.1871 | 0.9966 | 0.0003 | 0.0004 |
| *repo>hoRNAi* F | 0.7717 | 0.1978 | 0.2471 | 0.9370 | 0.0.0637 | 0.3588 |
| *elav>hoRNAi* M | 0.0001 | 0.0008 | 0.0088 | 0.2591 | 0.2193 | 0.9525 |
| *elav>hoRNAi* F | 0.0007 | 0.0143 | <0.0001 | 0.0002 | <0.0001 | 0.0104 |
| *repo>ho* M | 0.9692 | 0.9993 | 0.8159 | 0.9246 | 0.9679 | 0.3664 |
| *repo>ho* F | 0.9982 | 0.8254 | 0.9988 | 0.5083 | 0.0003 | 0.0381 |
| *elav>ho* M | 0.8652 | 0.9286 | 0.8572 | 0.6466 | 0.3156 | 0.5005 |
| *elav>ho* F | 0.8617 | 0.9176 | 0.0399 | 0.9985 | 0.1420 | 0.1013 |
| *repo>cncRNAi* M | 0.9846 | 0.4991 | 0.9977 | 0.2282 | 0.9645 | 0.9773 |
| *repo>cncRNAi* F | 0.9642 | 0.1212 | 0.9900 | 0.2005 | 0.9994 | 0.6502 |
| *elav>cncRNAi* M | 0.3102 | 0.9982 | 0.0.9867 | 0.2979 | 0.0221 | 0.5762 |
| *elav>cncRNAi* F | 0.9988 | 0.6181 | 0.9964 | 0.2736 | 0.9813 | 0.5760 |
| *repo>cnc* M | 0.1197 | 0.9777 | 0.4661 | 0.9877 | 0.0242 | 0.9622 |
| *repo>cnc* F | 0.0449 | 0.8793 | 0.2609 | 0.8426 | 0.0078 | 0.0117 |
| *elav>cnc* M | 0.9933 | 0.9833 | 0.9176 | 0.4060 | <0.0001 | <0.0001 |
| *elav>cnc* F | 0.5909 | 0.5229 | 0.3289 | 0.9417 | 0.2332 | 0.0456 |
| **Larvae-specific** | **Gal4**  **p-value** | **UAS**  **p-value** | **Gal4**  **p-value** | **UAS**  **p-value** | **Gal4**  **p-value** | **UAS**  **p-value** |
|  | **7 d** | | **14 d** | | **30 d** | |
| *repo>hoRNAi* M | 0.5896 | 0.7744 | 0.9849 | 0.8007 | 0.1545 | 0.4260 |
| *repo>hoRNAi* F | 0.8279 | 0.2914 | 0.5156 | 0.6052 | 0.9653 | 0.8191 |
| *elav>hoRNAi* M | 0.9144 | 0.9336 | 0.6805 | 0.9958 | 0.7437 | 0.5258 |
| *elav>hoRNAi* F | 0.6800 | 0.2043 | 0.4496 | 0.3507 | 0.6246 | 0.0702 |
| *repo>ho* M | 0.7766 | 0.9566 | 0.3889 | 0.9470 | 0.4655 | 0.9877 |
| *repo>ho* F | 0.9765 | 0.8326 | 0.7036 | 0.8276 | 0.6563 | 0.9614 |
| *elav>ho* M | 0.4946 | 0.8599 | 0.9383 | 0.9775 | 0.3660 | 0.3769 |
| *elav>ho* F | 0.6845 | 0.8055 | 0.4740 | 0.6478 | 0.3617 | 0.8135 |
| *repo>cncRNAi* M | 0.2001 | 0.2586 | 0.0.0862 | 0.7155 | 0.0045 | 0.6332 |
| *repo>cncRNAi* F | 0.7458 | 0.9985 | 0.7896 | 0.9973 | 0.3232 | 0.9994 |
| *elav>cncRNAi* M | 0.5682 | 0.9974 | 0.9756 | 0.2266 | 0.9173 | 0.3506 |
| *elav>cncRNAi* F | 0.9687 | 0.6307 | 0.9601 | 0.9979 | 0.9264 | 0.6246 |
| *repo>cnc* M | 0.9011 | 0.9974 | 0.9949 | 0.6996 | 0.9165 | 0.3116 |
| *repo>cnc* F | 0.4210 | 0.5096 | 0.9573 | 0.9968 | 0.0104 | 0.5862 |
| *elav>cnc* M | 0.6508 | 0.5974 | 0.7488 | 0.5649 | 0.5907 | 0.9397 |
| *elav>cnc* F | 0.7925 | 0.9645 | 0.9999 | 0.6003 | 0.8068 | 0.7078 |
| **Pupae-specific** | **Gal4**  **p-value** | **UAS**  **p-value** | **Gal4**  **p-value** | **UAS**  **p-value** | **Gal4**  **p-value** | **UAS**  **p-value** |
|  | **7 d** | | **14 d** | | **30 d** | |
| *repo>hoRNAi* M | 0.0051 | 0.0220 | 0.2691 | 0.1654 | 0.9730 | 0.3703 |
| *repo>hoRNAi* F | 0.7908 | 0.9257 | 0.2476 | 0.9919 | 0.6595 | 0.9578 |
| *elav>hoRNAi* M | 0.6678 | 0.0922 | 0.8653 | 0.9824 | 0.0143 | 0.2601 |
| *elav>hoRNAi* F | 0.3726 | 0.5034 | 0.0613 | 0.9745 | 0.7409 | 0.6041 |
| *repo>ho* M | 0.9692 | 0.9764 | 0.2087 | 0.5631 | 0.9849 | 0.8920 |
| *repo>ho* F | 0.9251 | >0.9999 | 0.4328 | 0.9860 | 0.5808 | 0.8232 |
| *elav>ho* M | 0.9248 | 0.1967 | 0.0047 | 0.0047 | 0.7860 | 0.1723 |
| *elav>ho* F | 0.8016 | 0.9210 | 0.9996 | 0.7119 | 0.9995 | 0.4391 |
| *repo>cncRNAi* M | 0.2670 | 0.6349 | 0.1132 | 0.5117 | 0.0027 | 0.0033 |
| *repo>cncRNAi* F | 0.7954 | 0.7307 | 0.8182 | 0.6653 | 0.9995 | 0.4380 |
| *elav>cncRNAi* M | 0.9139 | 0.9945 | 0.3783 | 0.0874 | 0.0002 | 0.8841 |
| *elav>cncRNAi* F | 0.8437 | 0.9267 | 0.8866 | 0.2531 | 0.9969 | 0.4852 |
| *repo>cnc* M | 0.6422 | 0.9194 | 0.8491 | 0.9754 | 0.1827 | 0.9438 |
| *repo>cnc* F | 0.2536 | 0.5371 | 0.0637 | 0.1712 | 0.0332 | 0.0462 |
| *elav>cnc* M | 0.2723 | 0.2723 | 0.0480 | 0.6540 | 0.2732 | 0.7719 |
| *elav>cnc* F | 0.8880 | 0.7115 | 0.4869 | 0.6196 | 0.6795 | 0.9864 |
| **Adult-specific** | **Gal4**  **p-value** | **UAS**  **p-value** | **Gal4**  **p-value** | **UAS**  **p-value** | **Gal4**  **p-value** | **UAS**  **p-value** |
|  | **7 d** | | **14 d** | | **30 d** | |
| *repo>hoRNAi* M | 0.8849 | 0.0486 | 0.1631 | 0.7364 |  |  |
| *repo>hoRNAi* F | 0.4592 | 0.3982 | 0.3318 | 0.1368 |  |  |
| *elav>hoRNAi* M | 0.9710 | 0.1752 | 0.0115 | 0.6750 | 0.0047 | 0.8903 |
| *elav>hoRNAi* F | 0.4773 | 0.2420 | 0.8141 | 0.6979 | 0.1097 | 0.0025 |
| *repo>ho* M | 0.1959 | 0.4422 | 0.5793 | 0.8571 |  |  |
| *repo>ho* F | 0.6250 | 0.9921 | 0.2893 | 0.2127 |  |  |
| *elav>ho* M | 0.9573 | 0.2900 | 0.1088 | 0.0415 | 0.1658 | 0.6604 |
| *elav>ho* F | 0.3589 | 0.9774 | 0.9019 | 0.7897 | 0.3068 | 0.0498 |
| *repo>cncRNAi* M | 0.0308 | 0.9994 | 0.0296 | 0.9936 |  |  |
| *repo>cncRNAi* F | 0.9369 | 0.7691 | 0.7824 | 0.4063 |  |  |
| *elav>cncRNAi* M | 0.5530 | 0.0215 | <0.0001 | 0.4082 | 0.5714 | <0.0001 |
| *elav>cncRNAi* F | 0.9334 | 0.8893 | 0.8830 | 0.2952 | <0.0001 | <0.0001 |
| *repo>cnc* M | 0.9764 | 0.0476 | 0.5417 | 0.9232 |  |  |
| *repo>cnc* F | 0.9374 | 0.2824 | 0.9919 | 0.8841 |  |  |
| *elav>cnc* M | 0.7564 | 0.5903 | 0.6701 | 0.0861 | 0.5353 | 0.9445 |
| *elav>cnc* F | 0.2093 | 0.7789 | 0.5899 | 0.9947 | 0.3393 | 0.0074 |
