# Supplementary material for: Changes in heme oxygenase level during development affect the adult life of Drosophila melanogaster
Source: Front Cell Neurosci. 2023 Oct 9;17:1239101. doi: 10.3389/fncel.2023.1239101 (PMC10591093; doi:10.3389/fncel.2023.1239101)
Supplement: Supplementary file 3 [file Table_3.DOCX]

**Supplementary Table 7.** Total activity of flies with modification of *ho* and *cnc* expression at specific developmental stage measured in minutes. Detailed statistics (p-value) for experimental flies compared with parental control strains (Gal4 and UAS).

|  | **Gal4 p-value** | **UAS p-value** |
| --- | --- | --- |
| **CHRONIC** |  |  |
| *repo>hoRNAi* | 0.5027 | 0.1356 |
| *elav>hoRNAi* | 0.0003 | <0.0001 |
| *repo>ho* | <0.0001 | <0.0001 |
| *elav>ho* | 0.9825 | 0.1951 |
| *repo>cncRNAi* | 0.0002 | <0.0001 |
| *elav>cncRNAi* | <0.0001 | <0.0001 |
| *repo>cnc* | <0.0001 | <0.0001 |
| *elav>cnc* | 0.1428 | <0.0001 |
| **LARVAE-specific** |  |  |
| *tubGAL80ts;repo>hoRNAi* | 0.6606 | <0.0001 |
| *tubGAL80ts;elav>hoRNAi* | 0.9584 | <0.0001 |
| *tubGAL80ts;repo>ho* | 0.0019 | 0.5203 |
| *tubGAL80ts;elav>ho* | 0.0539 | 0.6211 |
| *tubGAL80ts;repo>cncRNAi* | <0.0001 | 0.0003 |
| *tubGAL80ts;elav>cncRNAi* | 0.0160 | <0.0001 |
| *tubGAL80ts;repo>cnc* | <0.0001 | 0.0165 |
| *tubGAL80ts;elav>cnc* | 0.6640 | 0.6576 |
| **PUPAE-specific** |  |  |
| *tubGAL80ts;repo>hoRNAi* | 0.6060 | 0.0009 |
| *tubGAL80ts;elav>hoRNAi* | 0.2421 | <0.0001 |
| *tubGAL80ts;repo>ho* | 0.5239 | 0.3236 |
| *tubGAL80ts;elav>ho* | 0.0061 | 0.9345 |
| *tubGAL80ts;repo>cncRNAi* | 0.7989 | 0.2956 |
| *tubGAL80ts;elav>cncRNAi* | 0.2704 | 0.2874 |
| *tubGAL80ts;repo>cnc* | 0.0090 | 0.7044 |
| *tubGAL80ts;elav>cnc* | 0.5266 | <0.0001 |
| **ADULT-specific** |  |  |
| *tubGAL80ts;repo>hoRNAi* | 0.0143 | 0.4876 |
| *tubGAL80ts;elav>hoRNAi* | 0.2834 | 0.7484 |
| *tubGAL80ts;repo>ho* | <0.0001 | 0.4345 |
| *tubGAL80ts;elav>ho* | <0.0001 | 0.4963 |
| *tubGAL80ts;repo>cncRNAi* | 0.0008 | <0.0001 |
| *tubGAL80ts;elav>cncRNAi* | 0.0223 | 0.0004 |
| *tubGAL80ts;repo>cnc* | 0.0015 | 0.8062 |
| *tubGAL80ts;elav>cnc* | 0.0003 | 0.7371 |
