# Supplementary material for: Changes in heme oxygenase level during development affect the adult life of Drosophila melanogaster
Source: Front Cell Neurosci. 2023 Oct 9;17:1239101. doi: 10.3389/fncel.2023.1239101 (PMC10591093; doi:10.3389/fncel.2023.1239101)
Supplement: Supplementary file 4 [file Table_4.DOCX]

**Supplementary Table 8**. Detailed statistics for Fig 6. Sleep analysis for chronic modification of *ho* and *cnc* expression. Dunnett’s multiple comparison, experimental flies compared with parental strains (Gal4 and UAS, respectively).

| **Chronic** | **Gal4**  **p-value** | **UAS**  **p-value** | **Gal4**  **p-value** | **UAS**  **p-value** |
| --- | --- | --- | --- | --- |
|  | **Daytime sleep** | | **Nighttime sleep** | |
| *repo>hoRNAi* M | 0.2025 | 0.7606 | 0.3357 | 0.0021 |
| *elav>hoRNAi* M | 0.4248 | <0.0001 | <0.0001 | 0.0101 |
| *repo>ho* M | <0.0001 | <0.0001 | <0.0001 | <0.0001 |
| *elav>ho* M | 0.0027 | 0.9582 | 0.0025 | <0.0001 |
| *repo>cncRNAi* M | <0.0001 | <0.0001 | 0.0423 | 0.0056 |
| *elav>cncRNAi* M | <0.0001 | <0.0001 | <0.0001 | <0.0001 |
| *repo>cnc* M | <0.0001 | <0.0001 | <0.0001 | <0.0001 |
| *elav>cnc* M | 0.0204 | <0.0001 | <0.0001 | <0.0001 |
