# Supplementary material for: Changes in heme oxygenase level during development affect the adult life of Drosophila melanogaster
Source: Front Cell Neurosci. 2023 Oct 9;17:1239101. doi: 10.3389/fncel.2023.1239101 (PMC10591093; doi:10.3389/fncel.2023.1239101)
Supplement: Supplementary file 5 [file Table_5.DOCX]

**Supplementary Table 9.** Detailed statistics for qPCR data (for Fig. 7-10).

| Statistics for Figure 7 |  |  |
| --- | --- | --- |
| **CS 18 degrees** | ***ho*** | ***cnc*** |
| Larvae vs pupae | 0.6905 | 0.7302 |
| Larvae vs males | 0.0159 | 0.0286 |
| Larvae vs females | 0.0159 | 0.0286 |
| Pupae vs males | 0.0159 | 0.0159 |
| Pupae vs females | 0.0159 | 0.0159 |
| Males vs females | 0.8857 | 0.8857 |
| **CS 25 degrees** | ***ho*** | ***cnc*** |
| Larvae vs pupae | 0.0159 | 0.0317 |
| Larvae vs males | 0.0159 | 0.0159 |
| Larvae vs females | 0.0286 | 0.0317 |
| Pupae vs males | >0.9999 | 0.2222 |
| Pupae vs females | 0.9048 | 0.1508 |
| Males vs females | >0.9999 | 0.2163 |
| **CS 29 degrees** | ***ho*** | ***Cnc*** |
| Larvae vs pupae | 0.4127 | 0.1905 |
| Larvae vs males | 0.4127 | 0.0159 |
| Larvae vs females | 0.0556 | 0.0952 |
| Pupae vs males | 0.6857 | 0.6905 |
| Pupae vs females | 0.5556 | 0.9048 |
| Males vs females | 0.5556 | 0.6905 |
|  |  |  |
| Statistics for Figure 8 |  |  |
|  | **Gal4 p-value** | **UAS p-value** |
| **LARVAE-specific** |  |  |
| *tubGal80ts;repo>hoRNAi* L | 0.0507 | 0.1473 |
| *tubGal80ts;repo>hoRNAi* P | 0.5934 | 0.0225 |
| *tubGal80ts;repo>hoRNAi* M | 0.9121 | 0.2021 |
| *tubGal80ts;repo>hoRNAi* F | 0.9121 | 0.2021 |
| *tubGal80ts;elav>hoRNAi* L | 0.3594 | 0.0507 |
| *tubGal80ts;elav>hoRNAi* P | 0.2021 | >0.9999 |
| *tubGal80ts;elav>hoRNAi* M | 0.9121 | 0.2021 |
| *tubGal80ts;elav>hoRNAi* F | 0.9121 | 0.2021 |
| *tubGal80ts;repo>ho* L | 0.0507 | 0.1473 |
| *tubGal80ts;repo>ho* P | 0.7422 | 0.7422 |
| *tubGal80ts;repo>ho* M | >0.9999 | >0.9999 |
| *tubGal80ts;repo>ho* F | >0.9999 | >0.9999 |
| *tubGal80ts;elav>ho* L | 0.2021 | 0.0341 |
| *tubGal80ts;elav>ho* P | 0.4661 | 0.5934 |
| *tubGal80ts;elav>ho* M | 0.5934 | 0.0225 |
| *tubGal80ts;elav>ho* F | 0.2721 | 0.0738 |
| **PUPAE-specific** |  |  |
| *tubGal80ts;repo>hoRNAi* P | 0.4661 | >0.9999 |
| *tubGal80ts;repo>hoRNAi* M | 0.1051 | >0.9999 |
| *tubGal80ts;repo>hoRNAi* F | 0.0738 | 0.5934 |
| *tubGal80ts;elav>hoRNAi* P | >0.9999 | >0.9999 |
| *tubGal80ts;elav>hoRNAi* M | 0.0146 | 0.3594 |
| *tubGal80ts;elav>hoRNAi* F | 0.2021 | 0.9121 |
| *tubGal80ts;repo>ho* P | 0.0225 | 0.2721 |
| *tubGal80ts;repo>ho* M | 0.7422 | 0.0507 |
| *tubGal80ts;repo>ho* F | >0.9999 | >0.9999 |
| *tubGal80ts;elav>ho* P | 0.0341 | 0.9121 |
| *tubGal80ts;elav>ho* M | 0.9121 | >0.9999 |
| *tubGal80ts;elav>ho* F | 0.3594 | 0.7422 |
| **ADULT-specific** |  |  |
| *tubGal80ts;repo>hoRNAi* M | 0.0146 | 0.3594 |
| *tubGal80ts;repo>hoRNAi* F | 0.0738 | 0.1053 |
| *tubGal80ts;elav>hoRNAi* M | 0.3594 | 0.3594 |
| *tubGal80ts;elav>hoRNAi* F | >0.9999 | >0.9999 |
| *tubGal80ts;repo>ho* M | 0.0146 | 0.3594 |
| *tubGal80ts;repo>ho* F | 0.0341 | 0.2021 |
| *tubGal80ts;elav>ho* M | 0.1473 | 0.0507 |
| *tubGal80ts;elav>ho* F | 0.1473 | 0.0507 |
|  |  |  |
| Statistics for Figure 9 |  |  |
| ***ho* compensation** | **Gal-4**  **p-value** | **UAS**  **p-value** |
| *repo>hoRNAi* head | 0.0002 | 0.0022 |
| *repo>hoRNAi* brain + fat body | 0.3146 | >0.9999 |
| GMR*>hoRNAi* brain + fat body | 0.5931 | 0.1680 |
| *repo>hoRNAi* retina | 0.018 | 0.0079 |
| GMR>*hoRNAi* retina | 0.0286 | 0.0023 |
|  |  |  |
| Statistics for Figure 10 | **Gal-4**  **p-value** | **UAS**  **p-value** |
| *repo>hoRNAi* F | >0.9999 | >0.9999 |
| *elav>hoRNAi* L | 0.0225 | 0.2721 |
| *elav>hoRNAi* P | 0.0225 | 0.2721 |
| *elav>hoRNAi* M | >0.9999 | >0.9999 |
| *elav>hoRNAi* F | 0.0073 | 0.0532 |
| *repo>ho* L | >0.9999 | 0.0507 |
| *repo>ho* P | 0.0225 | 0.2721 |
| *repo>ho* M | 0.0216 | 0.0216 |
| *repo>ho* F | 0.0197 | 0.0236 |
| *elav>ho* L | 0.1473 | >0.9999 |
| *elav>ho* P | 0.0738 | 0.5934 |
| *elav>ho* M | 0.0026 | 0.0118 |
| *elav>ho* F | 0.0236 | 0.0482 |
| *repo>cncRNAi* L | 0.5934 | 0.2721 |
| *repo>cncRNAi* P | 0.0738 | 0.1053 |
| *repo>cncRNAi* M | 0.0145 | 0.0181 |
| *repo>cncRNAi* F | 0.0275 | 0.0025 |
| *elav>cncRNAi* L | 0.0146 | 0.3594 |
| *elav>cncRNAi* P | 0.2021 | 0.0341 |
| *elav>cncRNAi* M | 0.8772 | 0.6333 |
| *elav>cncRNAi* F | 0.2458 | 0.0063 |
| *repo>cnc* L | 0.1473 | 0.0507 |
| *repo>cnc* P | 0.3594 | 0.0146 |
| *repo>cnc* M | 0.0352 | 0.0233 |
| *repo>cnc* F | 0.0091 | 0.0049 |
| *elav>cnc* L | 0.0146 | 0.3594 |
| *elav>cnc* P | 0.2021 | 0.0341 |
| *elav>cnc* M | 0.0214 | 0.0389 |
| *elav>cnc* F | 0.0230 | 0.0491 |
